# Structure of the human sodium leak channel NALCN in complex with FAM155A

**DOI:** 10.1038/s41467-020-19667-z

**Published:** 2020-11-17

**Authors:** Jiongfang Xie, Meng Ke, Lizhen Xu, Shiyi Lin, Jin Huang, Jiabei Zhang, Fan Yang, Jianping Wu, Zhen Yan

**Affiliations:** 1 Key Laboratory of Structural Biology of Zhejiang Province, School of Life Sciences, Westlake University, 310024 Hangzhou, Zhejiang China; 2 Westlake Laboratory of Life Sciences and Biomedicine, 310024 Hangzhou, Zhejiang China; 3 Institute of Biology, Westlake Institute for Advanced Study, 310024 Hangzhou, Zhejiang China; 4 Department of Biophysics and Kidney Disease Center, First Affiliated Hospital, Zhejiang University School of Medicine, 310058 Hangzhou, China

**Keywords:** Ion channels in the nervous system, Cryoelectron microscopy

## Abstract

NALCN, a sodium leak channel expressed mainly in the central nervous system, is responsible for the resting Na^+^ permeability that controls neuronal excitability. Dysfunctions of the NALCN channelosome, NALCN with several auxiliary subunits, are associated with a variety of human diseases. Here, we report the cryo-EM structure of human NALCN in complex with FAM155A at an overall resolution of 3.1 angstroms. FAM155A forms extensive interactions with the extracellular loops of NALCN that may help stabilize NALCN in the membrane. A Na^+^ ion-binding site, reminiscent of a Ca^2+^ binding site in Ca_v_ channels, is identified in the unique EEKE selectivity filter. Despite its ‘leaky’ nature, the channel is closed and the intracellular gate is sealed by S6_I_, II-III linker and III-IV linker. Our study establishes the molecular basis of Na^+^ permeation and voltage sensitivity, and provides important clues to the mechanistic understanding of NALCN regulation and NALCN channelosome-related diseases.

## Abstract (web summary)

NALCN, a sodium leak channel, plays a key role in regulating the resting membrane potential and controlling neuronal excitability. Here the authors report a cryo-EM structure of human NALCN in complex with FAM155A, that with complementary functional analyses provide insights on its ion selectivity, voltage sensing and specific interactions with auxiliary subunits.

## Introduction

The membrane potential across the cell membrane is essential for signal transduction in excitable cells such as neurons and muscle cells^1^. In neurons, the resting membrane potential (RMP) is approximately −70 mV, considerably depolarized as compared to the equilibrium potential of K^+^ of −90 mV. The depolarization is mostly attributable to resting Na^+^ permeability, which is important for the regulation of neuronal excitability^2,3^. The newly identified ion channel NALCN (Na^+^ leak channel, non-selective) has a key role in regulating RMP and controlling neuronal excitability. It is primarily expressed in the central nervous system and is mainly responsible for neuronal tetrodotoxin (TTX)-resistant Na^+^ leak conductance^4^.

NALCN consists of a single polypeptide chain of 24 transmembrane helices (TM) that form four homologous functional repeats connected by intracellular linkers. The topology of NALCN is similar to that of voltage-gated sodium (Na_v_) channels and voltage-gated calcium (Ca_v_) channels (4 × 6 TM). Each functional repeat of NALCN contains six TMs (S1-S6), with S1-S4 corresponding to the voltage-sensing domain (VSD) in Na_v_/Ca_v_. S5, S6, and intervening segments, including the pore helices and selectivity filter (SF) from all four repeats, constitute the ion-conducting pore. NALCN shares less than 20% identity with any known Na_v_ or Ca_v_ channel, and has been classified as a member of a new subclass of the 4 × 6 TM ion channel family^5^. Compared to Na_v_ and Ca_v_ channels, NALCN contains fewer positively charged residues (arginine or lysine) on S4 segments. In addition, NALCN has a unique ion SF with EEKE residues in the four repeats, which is different from the SFs of Na_v_ channels (DEKA) and Ca_v_ channels (EEEE/EEDD)^5^.

NALCN is associated with several auxiliary subunits, including UNC80, UNC79, and FAM155A (Family with sequence similarity 155, member A). Together, they form a large protein complex termed the NALCN channelosome. Notably, these auxiliary subunits are unique to NALCN and exhibit no sequence homology to the auxiliary subunits of any other channel. Both UNC80 and UNC79 are large proteins that contribute to the neuronal localization and stabilization of NALCN in *C. elegans* and *D. melanogaster*, although their own functional domains and subcellular localization are largely unclear^6–8^. UNC80 directly interacts with NALCN and acts as a scaffold protein for UNC79^9,10^. FAM155A, also named NLF-1 in *C. elegans*, was reported to be an endoplasmic reticulum (ER) resident protein that acts as a chaperone to facilitate the folding and promote the axon delivery of NALCN^11^. In humans, FAM155B, a FAM155A homolog, can functionally substitute for FAM155A, and is likely a component of the NALCN channelosome^12^. In addition, NALCN channelosome activity is regulated by the Src family of tyrosine kinases (SFKs) and several GPCRs such as the M3 muscarinic receptor (M3R) through direct interactions^13^. The NALCN channel is also activated by neuropeptides, including substance P and neurotensin, through the SFK-dependent pathway in mouse hippocampal neurons^14^. How NALCN interacts with and is regulated by its auxiliary subunits remains largely unclear.

NALCN is evolutionally conserved in both invertebrate and vertebrate species. For example, NALCN homologs have been identified in invertebrate species such as snails and *C. elegans*, in which Na_v_ homologs were not found^5,15^. This indicates that NALCN evolved earlier than Na_v_ channels across species. NALCN is highly conserved in mammals, with more than 96% sequence identity among humans, mice, rats, rabbits and bovines. Human NALCN also shares at least 44% identity and 56% similarity with its homologs α1U in *D. melanogaster* and NCA-1/2 in *C. elegans*.

In addition to its role in regulating neuronal excitability, NALCN is also important in many fundamental physiological processes, such as motor function, pain sensitivity and circadian rhythm. For example, NALCN mutant mice die within 24 h after birth due to disrupted respiratory rhythm^4^. Overexpression of NALCN in highveld mole-rats decreases the detection of painful substances by nociceptors^16^. Reduced NALCN expression in *Drosophila* leads to changes in behavioral circadian rhythms and sensitivity to anesthetics^17,18^. In humans, NALCN variants are linked to a variety of diseases known as NALCN channelopathies, including congenital contractures of the limbs and face, hypotonia and developmental delay (CLIFAHDD)^19–21^, psychomotor retardation and characteristic facies (IHPRF)^22,23^, infantile neuroaxonal dystrophy (INAD)^24^, cervical dystonia, schizophrenia and bipolar disorder^25–27^. There are also many diseases related to dysfunctions of the auxiliary subunits of NALCN^28,29^.

Despite its physiological and pathological importance, the biophysical properties of the voltage sensitivity and ion selectivity of NALCN are still under debate. Electrophysiological studies on NALCN have been performed in heterologous expression systems such as HEK293 cells, *X. laevis* oocytes and the neuronal cell line NG108-15^4,12,30^. NALCN expressed in HEK293 cells was reported to exhibit a linear current–voltage relationship, suggesting that NALCN is a voltage-independent ion channel^4^. However, other studies suggest that the VSDs of NALCN can exhibit a broader range of gating behaviors and coexpression of NALCN, UNC79, UNC80, and FAM155A resulted in voltage-dependent NALCN currents^12,30^. NALCN was originally reported to be non-selective and permeable to Na^+^, K^+^, and Ca^2+^ ions^4^. Nevertheless, NALCN was recently reported to be selective only for monovalent cations and to be blocked by extracellular divalent cations^12^.

To better understand the functional properties and mechanisms of NALCN, we sought to determine the structure of NALCN in complex with its auxiliary subunits. Here, we report the cryo-EM structure of human NALCN in complex with FAM155A at an overall resolution of 3.1 Å. Our structure, along with electrophysiology and molecular dynamics (MD) simulation data, provides important insights into the structure, function and regulation of the NALCN channelosome. It is to our attention that as we were submitting the manuscript, two similar works were reported elsewhere^31,32^. These back-to-back works mutually verified the main conclusions and ours also provides several novel points, such as unique lipid binding in repeat I and Na^+^ binding sites analysis in EEKE SF, as will be described below.

## Results

### Functional characterization of NALCN by electrophysiology

We employed patch-clamp recordings in HEK293 cells to characterize the electrophysiological properties of NALCN. The current elicited in response to voltage steps was very small and appeared to be indistinguishable from the mock result when NALCN was expressed alone in HEK293 cells (Supplementary Fig. 1a). In contrast, coexpression of NALCN with UNC79, UNC80, and FAM155A dramatically enhanced the current, which was largely inhibited by 1 mM verapamil, implying that the current measured was mainly mediated by NALCN (Supplementary Fig. 1b). We also recorded the kinetics of the voltage-sensitive NALCN current: hyperpolarization voltages elicited a large inward current with inactivation, while depolarization voltages activated the current with an exponential time course before reaching the steady state (Supplementary Fig. 1b). These observations indicate that the auxiliary subunits are essential for a functional NALCN, which is consistent with previous studies^12,30^. The electrophysiological properties of NALCN suggest that it tends to maintain the RMP in a manner reminiscent of Le Chatelier’s principle in chemical equilibria. In comparison, Na_v_ channels always show rapid current activation and inactivation for fast initiating action potentials^33^, while certain K_v_ channels such as KCNQ channels show slow activation kinetics to avoid fast repolarization^34^.

### Structure determination of the human NALCN-FAM155A complex

**Fig. 1:**
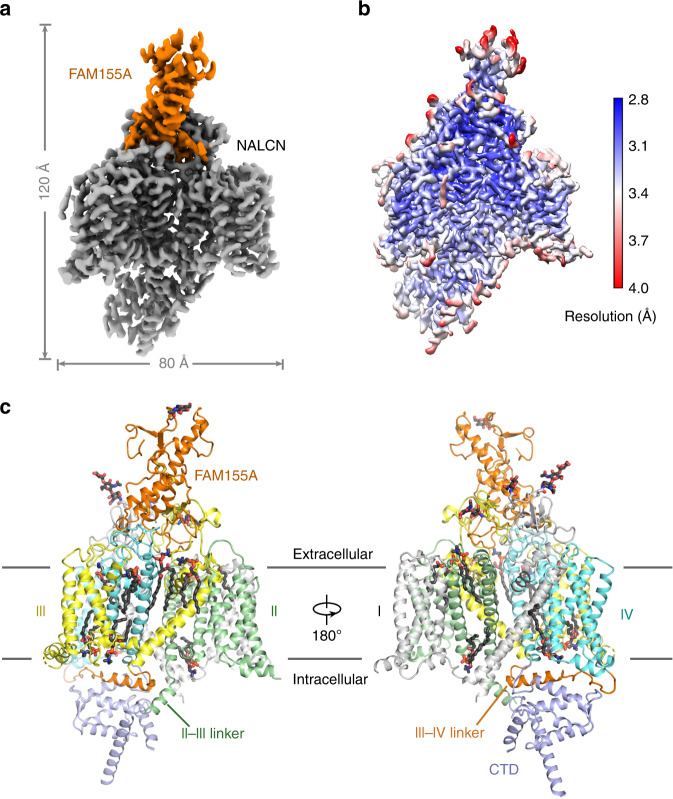
Cryo-EM structures of human NALCN in complex with FAM155A. **a** Cryo-EM map of human NALCN in complex with FAM155A. NALCN and FAM155A are colored gray and orange, respectively. The map was contoured at 0.8 threshold level in ChimeraX^70^. **b** Local resolution map estimated by cryoSPARC^52^ and generated in Chimera^57^. **c** Overall structure of the NALCN-FAM155A complex presented in two side views. The protein structure is shown in cartoon. NALCN is domain colored with repeats I, II, III, IV, and the CTD in light gray, green, yellow, cyan, and slate, respectively. The III-IV linker and FAM155A are colored orange. Lipid molecules and the sugar moieties at the glycosylation sites are shown as sticks. The color schemes are applied in all figures. All structure figures were prepared with PyMOL^71^.

Based on our functional characterizations, we focused on the coexpression of the four components in HEK293 cells. Unfortunately, we were unable to obtain a stable complex of the four components. Instead, a stable and homogeneous subcomplex of NALCN and FAM155A was obtained (Supplementary Fig. 2a). The peak fractions from gel filtration purification were pooled and concentrated to approximately 8.5 mg/ml for cryo-EM sample preparation. The EM images were collected on a Titan Krios electron microscope operating at 300 kV and equipped with a K3 direct detector and a GIF Quantum energy filter. After a few rounds of two-dimensional (2D) and 3D classifications, about sixty-five thousand particles were selected, which yielded a final reconstruction map with an overall resolution of 3.1 Å (Fig. 1 and Supplementary Table 1, Supplementary Figs. 2, 3). Local masks of the extracellular and intracellular regions were applied to further improve the local resolutions to 2.9 and 3.1 Å, respectively.

The density of the final reconstruction is of high quality. Most side chains were clearly resolved, allowing for accurate model building (Fig. 1a, b and Supplementary Figs. 4, 5). The model of NALCN was built starting from a homology model based on Ca_v_1.1 (PDB: 5GJV), whereas FAM155A was built de novo. In total, 1278 and 182 residues were built for NALCN and FAM155A, respectively. The complex structure was confirmed by cross-linking mass spectrometry analysis (Supplementary Fig. 6). Three glycosylation sites (Asn210, Asn216, and Asn1064) were identified on NALCN and one glycosylation site (Asn217) on FAM155A (Supplementary Fig. 5c). Four disulfide bonds were recognized in the extracellular loops (ECLs) of NALCN and six in FAM155A (Supplementary Fig. 5c). The glycosylation sites and disulfide bonds in turn further verified the accuracy of the structure.

**Fig. 2:**
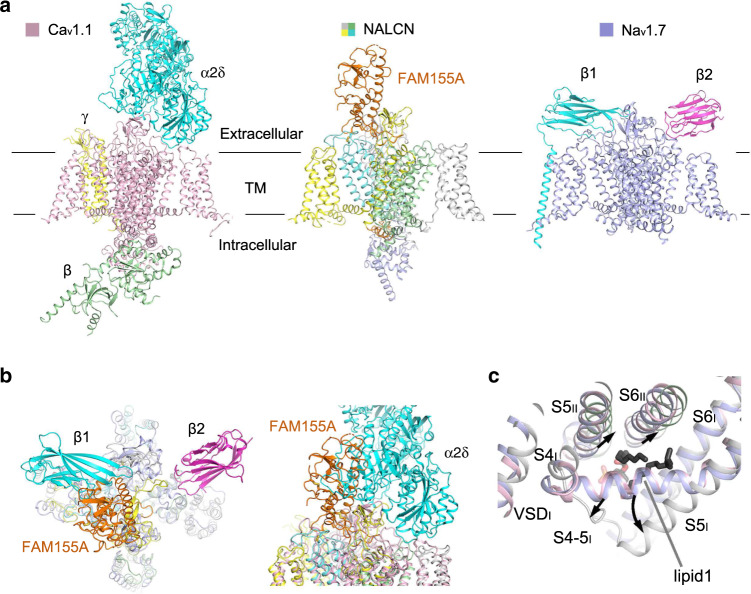
Structural comparisons between NALCN and Na_v_/Ca_v_ channels. **a** Overall structures of human NALCN, human Na_v_1.7 (PDB: 6J8I), and rabbit Ca_v_1.1 (PDB: 6JP5). The ion conducting subunits of Na_v_1.7 and Ca_v_1.1 are colored slate and light pink, respectively. The auxiliary subunits β1 and β2 of Na_v_1.7 are colored cyan and magenta, and the auxiliary subunits α2δ, β, γ of Ca_v_1.1 are colored cyan, yellow, and green, respectively. **b** FAM155A adopts a unique binding site on NALCN that differs from the auxiliary subunits of Na_v_/Ca_v_ channels. The structure of NALCN is superimposed with Na_v_1.7 or Ca_v_1.1 based on the ion-conducting subunit of each complex. Na_v_1.7 is shown in an extracellular view (left), and Ca_v_1.1 is shown in a side view (right). **c** Structural features in repeat I and a unique lipid-binding site of NALCN. The S4-5_I_ linker of NALCN is a loop instead of a helix in Na_v_1.7/Ca_v_1.1. S5_I_ in NALCN is extended three-helix turns into the cytosol compared to that of Na_v_1.7/Ca_v_1.1. An identified lipid molecule in NALCN occupies a position that corresponds to the S4-5 helix in Na_v_1.7/Ca_v_1.1. Local structural deviations around the lipid between NALCN and Na_v_1.7/Ca_v_1.1 are indicated by black arrows.

The overall structure of NALCN resembles those of eukaryotic Na_v_ and Ca_v_ channels (Figs. 1c and 2). We selected human Na_v_1.4 (PDB: 6AGF) and rabbit Ca_v_1.1 (PDB: 6JP5) as representative Na_v_ and Ca_v_ channels for comparisons with NALCN^35,36^. The structures of human Na_v_1.7 (PDB: 6J8I), rat Na_v_1.5 (6UZ3) and American cockroach Na_v_PaS (6A95) were also selected for specific discussions^37–39^. The structures of NALCN can be superimposed to the α subunit of Na_v_1.4 and α1 subunit Ca_v_1.1 with root-mean-square deviation (r.m.s.d.) of 2.99 Å over 760 Cα atoms and 3.894 Å over 905 Cα atoms, respectively. In addition to the four homologous repeats, a C-terminal domain (CTD) after repeat IV, which was observed in the structures of Ca_v_1.1 and Na_v_PaS but not in human Na_v_ channels, was also clearly resolved in the structure of NALCN (Fig. 1c). NALCN has no N-terminal domain (NTD) before repeat I, which always exists in Na_v_ channels^38–40^.

NALCN has several unique features that distinguish it from Na_v_ and Ca_v_ channels. FAM155A adopts a distinct binding interface on NALCN, which is different from those between the ion-conducting α subunit of Na_v_ and Ca_v_ channels and their auxiliary subunits (Fig. 2b). Moreover, the S4-5_I_ linker unexpectedly forms a flexible loop instead of a juxtamembrane helix as seen in the other three repeats of NALCN and other Na_v_/Ca_v_ structures (Fig. 2c). Meanwhile, S5_I_ elongates to the cytosol by three-helix turns compared to that of Na_v_/Ca_v_ channels. Notably, the unique structure of S4-5_I_ and S5_I_ results in a cavity that accommodates a lipid, which may contribute to the regulation of NALCN (Fig. 2c and Supplementary Fig. 4b). The local structure around the lipid is very different between NALCN and Na_v_/Ca_v_ channels. Intriguingly, the lipid sits in a position that is occupied by S4-5_I_ linker in Na_v_/Ca_v_ structures (Fig. 2c). In addition, a short helix designated II-III linker, which is connected to S6_II_ by a short loop, was observed in the structure of NALCN (Fig. 1c).

### Specific interactions between NALCN and FAM155A

**Fig. 3:**
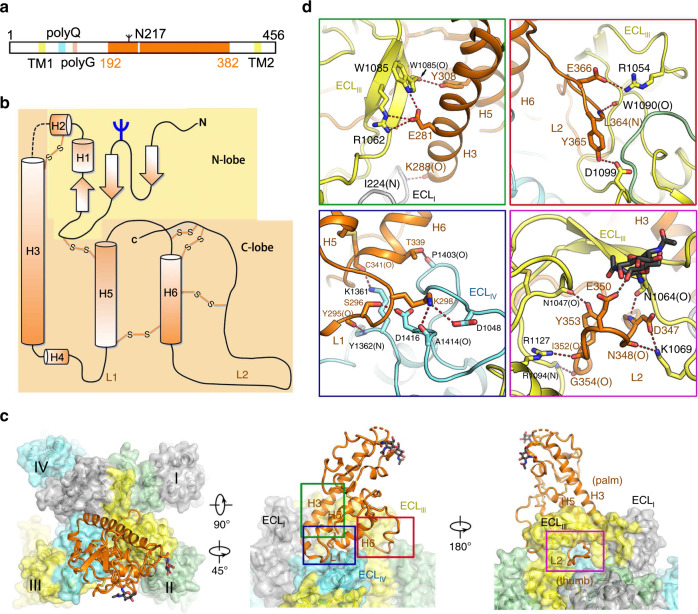
Specific interactions between NALCN and FAM155A. **a** One-dimensional (1D) schematic view of FAM155A. The region that was resolved in the current structure is colored orange. The position of the glycosylation site and predicted two transmembrane helices, polyQ, and polyG motifs, are labeled. **b** 2D topological structure of FAM155A. **c** Interaction interface between NALCN and FAM155A. NALCN is shown in surface and FAM155A is shown in cartoons. Details in the rectangle boxes are presented in **d**. **d** Zoom in views of each interaction interface. The electrostatic interactions are indicated by red dashed lines. The residues from NALCN and FAM155A are labeled black and orange, respectively. Please also refer to Supplementary Table 2 for a summary of the interactions between NALCN and FAM155A.

Human FAM155A contains 456 amino acids, and residues 192–382 were resolved in our structure except for a short loop from residues 250–258 (Fig. 3a). The resolved structure of FAM155A contains two lobes, designated the N-lobe and C-lobe, stabilized by six disulfide bonds. The N-lobe consists of three short β strands and two short α helices (H1–H2), while the C-lobe contains four α helices (H3–H6) (Fig. 3b). The overall structure of FAM155A is different from that of any auxiliary subunit in Na_v_ or Ca_v_ channels (Fig. 2b, Supplementary Fig. 7a). A search of the protein data bank using the DALI server^41^ revealed that the C-lobe of FAM155A contains a fold (similarity *Z*-score 4.3) similar to the structure of the frizzled-like cysteine-rich domain (CRD) of receptor tyrosine kinase MuSK (PDB: 3HKL), which had 84 Cα atoms aligned to FAM155A with an r.m.s.d. of 2.8 Å (Supplementary Fig. 7b). Moreover, three disulfide bonds are structurally conserved between the two structures (Supplementary Fig. 7b).

Only the C-lobe of FAM155A is involved in the interaction with NALCN. FAM155A sits above the pore domain of repeat IV of NALCN and forms extensive interactions with the ECL_I_, ECL_III_, and ECL_IV_ of NALCN (Fig. 3c and Supplementary Table 2). The loop in FAM155A connecting H4 and H5 and a hairpin loop after H6 are designated L1 and L2, respectively (Fig. 3b). Notably, L2 of FAM155A inserts deeply into a dome formed by ECL_III_ of NALCN. The H3, H5, H6 helices, and L2 of FAM155A resemble the palm and thumb of a hand, respectively, holding ECL_III_ of NALCN tightly (Fig. 3c). Detailed analysis of the interaction interfaces revealed extensive salt bridges and hydrogen bonds between NALCN and FAM155A (Fig. 3d and Supplementary Table 2). Unexpectedly, the sugar moiety linked to Asn1064 on ECL_III_, seems also contributed to the interaction by potentially forming a hydrogen bond with E350 on FAM155A (Fig. 3d). Electrostatic surface analysis revealed that two surfaces between NALCN and FAM155A are electrically complementary, ensuring a favorable interaction stability (Supplementary Fig. 7c). Sequence alignment among NALCN and Na_v_/Ca_v_ channels revealed that most of the interface residues in NALCN, including Asn1064, are not conserved among other Na_v_/Ca_v_ channels, indicating the high binding specificity of FAM155A towards NALCN, but not other channels (Supplementary Fig. 8). On the other hand, the key residues in the interaction interface are highly conserved among NALCN and FAM155A from different species, indicating that the interaction mode is evolutionarily conserved (Supplementary Fig. 9). Furthermore, the key residues mediating complex formation in FAM155A are also highly conserved in FAM155B, suggesting FAM155B may also be able to form a stable complex with NALCN in vivo (Supplementary Fig. 9). This notion is supported by the finding that FAM155A can be functionally substituted by human FAM155B^12^.

As UNC79 and UNC80 were not resolved in the structure, we then asked whether coexpression of NALCN and FAM155A alone was also able to recapitulate measurable NALCN currents. Interestingly, we readily recorded a large current in response to voltage steps when NALCN and FAM155A were cotransfected (Supplementary Fig. 1c). The current–voltage curve of NALCN coexpressed with either all three auxiliary subunits or with FAM155A alone was nonlinear (Supplementary Fig. 1d). The current response of NALCN with FAM155A exhibited similar voltage sensitivity to that of NALCN coexpressed with three auxiliary subunits, as the apparent gating charge (*Z*_app_) was similar (1.22 ± 0.18 *e*_0_ and 1.25 ± 0.55 *e*_0_, respectively) (Supplementary Fig. 1d, e). However, the *G*–*V* curve of NALCN coexpressed with three auxiliary subunits was largely shifted towards the hyperpolarization voltage compared to NALCN with FAM155A alone (*V*_1/2_: −65.07 ± 2.11 and −44.66 ± 1.91 mV, respectively) (Supplementary Fig. 1e). Moreover, we observed no inactivation of the steady-state current upon setting the prepulse potential to different levels when NALCN was coexpressed with auxiliary subunits (Supplementary Fig. 1f, g), which is consistent with the physiological role of NALCN as a leaky sodium channel. The observation that FAM155A alone is also able to recapitulate NALCN function is different from several recent studies^31,42^.

### Structural basis for the voltage dependence of NALCN

**Fig. 4:**
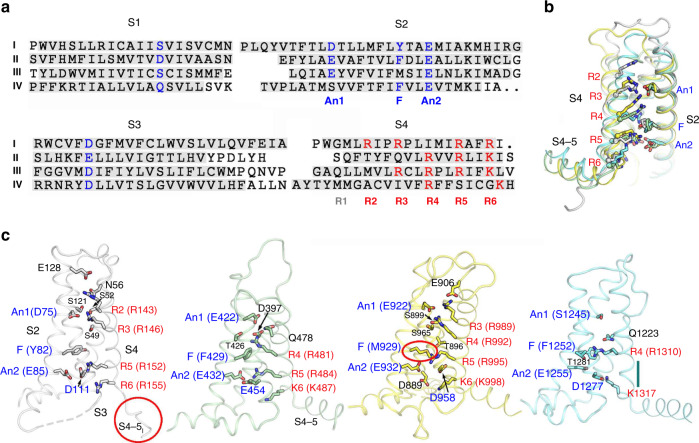
Structural features of the VSDs of NALCN. **a** Structure-based sequence alignment of the four VSDs. The boundaries for the S1 to S4 segments are shaded gray. The gating charges (GCs) in the S4 helices are colored red. The residues that correspond to the charge transfer center (CTC) on S2 and S3 helices as well as the polar or acidic residues on S1 that coordinate GCs are highlighted in blue. **b** All four VSDs adopt up or depolarized state. The four VSDs are superimposed relative to CTC and An1 on S2. For visual clarity, the S1 segments are omitted. **c** Structural details of each VSD. The GCs, CTCs, and the polar or acidic residues participating in GC coordination are shown in sticks. The GCs are labeled in red. The S4-5_I_ loop and non-conserved aromatic residues in the CTC of repeat III are highlighted by red circles. The short 3_10_ helix turn in repeat IV is indicated by a dark green line.

Our structural study has provided an opportunity to understand the voltage sensitivity of NALCNs. Detailed analysis of the VSDs of NALCN suggests that they possess several key features shared among functional VSDs in Na_v_/Ca_v_ channels. For instance, each VSD preserves the positively charged residues known as gating charges (GCs) in a pattern of occurring every three amino acids in the S4 segments. As usual, we define the position of the last GC in each S4 as R6^43^. NALCN has two to four GCs in each repeat distributed at positions R2–R6 (Fig. 4). The last GC of repeat IV is at a position that has one residue shift to R6, a phenomenon also observed in other channels such as Ca_v_1.1^43^. Moreover, the residues of the charge transfer center (CTC) which consists of a negatively charged residue (An2) and an aromatic occluding residue (F) on S2, and a negatively charged residue on S3, are highly conserved in NALCN except for the occluding residue on VSD_III_. The An1 sites, which lie seven residues ahead of the occluding residues on S2 segments of NALCN, are mainly negatively charged or polar residues, similar to those of Na_v_/Ca_v_ channels (Fig. 4a, b). In addition, previous structural studies on Na_v_ channels identified several negatively charged or polar residues on S1 that may also have roles in GC transfer^35^. These residues were also observed on NALCN (Fig. 4a). When the four VSDs are superimposed relative to An1 and CTC, all R4 residues are above the occluding residues on S2, reminiscent of up or depolarized states (Fig. 4b and Supplementary Fig. 10). Further comparisons with Na_v_PaS whose VSD_III_ and VSD_IV_ are in a potential resting state, revealed obvious upward movement of the S4 segments from Na_v_PaS to NALCN, confirming the depolarized conformations of VSDs in NALCN (Supplementary Fig. 10).

The voltage sensitivity of NALCN, however, seems to be lower than that of Na_v_/Ca_v_ channels (Supplementary Fig. 1). Several unique structural features of NALCN may lead to its relatively weak voltage sensitivity. First, the occluding residue on S2 in repeat III is replaced by a methionine (Met929), instead of a conserved phenylalanine or tyrosine (Fig. 4c). The occluding residue of the CTC is reported to be crucial for the GC transfer during voltage sensing^44^. Second, the S4 segments of VSDs are usually formed as 3_10_ helices, whereas in repeat IV the S4 largely relaxes to a regular α helix. The only 3_10_ helix turn in S4_IV_, which is at position R5, is a serine instead of a GC. Therefore, VSD_III_ and VSD_IV_ may contribute little to the voltage sensitivity, due to lack of occluding residue on S2_III_ and GCs and 3_10_ helices on S4_IV_, respectively. This structure-based speculation is consistent with a reported functional study, in which neutralization of the GCs in repeat III/IV has little effect on voltage sensitivity^12^. In contrast, neutralization of the GCs R146 + R152 in repeat I or R481 + R484 or R481 + K487 in repeat II led to significant changes in voltage sensitivity^12^. Third, the upper GCs (R2-R4) in repeat I-III form more extensive hydrogen bond interactions with surrounding negatively charged or polar residues than their counterparts in Na_v_/Ca_v_ channels (Fig. 4c). During the GC transfer process, the GCs break old interactions with surrounding residues and form new interactions with other residues, accompanied by a sliding movement along the S4 segment. The extensive interactions of the GCs and surrounding residues in NALCN may require the VSDs to overcome a higher energy barrier to undergo conformational changes, weakening the voltage sensitivity. Fourth, as S4-5 linkers are supposed to be a key transducer from VSDs to the ion-conducting pore during voltage sensing gating in VGICs^45^, the relaxation to a flexible linker of S4-5_I_ may hinder the effective transduction of the electromechanical coupling of repeat I in NALCN. Altogether, these structural variations in NALCN may contribute to its low voltage sensitivity.

### EEKE selectivity filter in NALCN

**Fig. 5:**
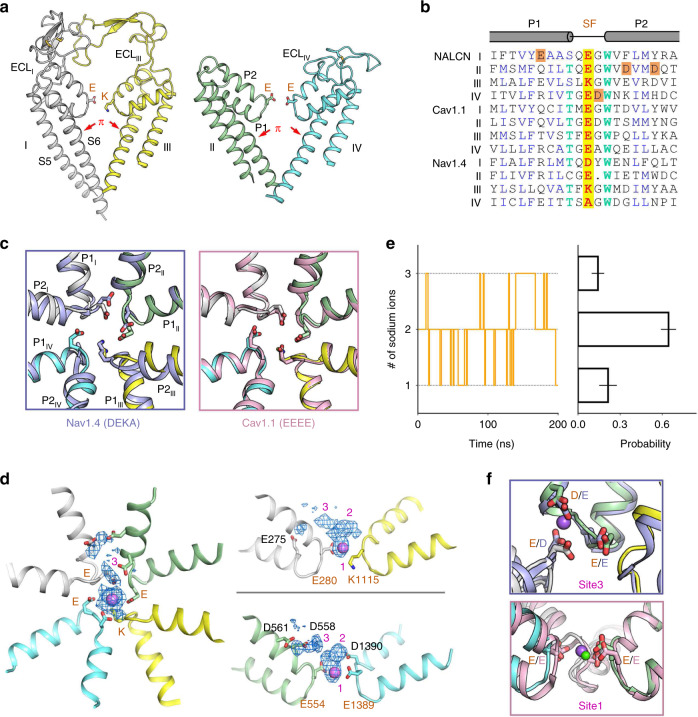
The EEKE selectivity filter of NALCN. **a** Overall structure of the pore domain of NALCN. Side views of the pore domain from the diagonal repeats are shown. The EEKE residues in the selectivity filter (SF) are shown in stick. Repeat I and repeat III adopt large extracellular loops, reminiscent of Ca_v_1.1. A π helix appears in each S6 segment and is indicated by red arrows. **b** Sequence alignment of the SF and the connecting pore helices among NALCN, Ca_v_1.1, and Na_v_1.4. The critical EEKE (E280, E554, K1115, and E1389) residues in NALCN and the corresponding residues in Ca_v_1.1 (EEEE) and Na_v_1.4 (DEKA) are shaded yellow. The invariant Trp residues and the highly conserved Thr residues are colored light green. The acidic residues that may help to coordinate cations in NALCN are shaded orange. **c** Structure comparisons of the SF between NALCN and Na_v_1.4 (*left*) or Ca_v_1.1 (*right*). The critical residues in the SF are shown in the stick. **d** Top view and two side views of sodium probability density generated from pore domain equilibration. Three potential Na^+^ binding sites were identified and labeled in purple. The most stable binding site (Site1) is signposted by a Na^+^ ion (magenta sphere). Please also refer to Supplementary Movie for the illustrative trajectory of equilibration. **e** Ion numbers within 4 Å of SF residues (EEKE) over time (left) and the probability statistics (right). The statistics represent the results of three independent simulations. **f** Structural comparison of Site 3 (EED motif) in NALCN and the Na^+^ binding site (DEE motif) in Na_v_1.4 (top) and of Site1 in NALCN and a Ca^2+^ binding site in Ca_v_1.1 (bottom). Na^+^ and Ca^2+^ are shown as purple and green spheres, respectively.

Like all reported Na_v_/Ca_v_ structures, the pore domain of NALCN is formed by the S5 and S6 helices of the four repeats, and the SF of NALCN is supported by two-pore helices (P1 and P2) that intervene between S5 and S6 (Fig. 5a). The key residues in the SF of NALCN responsible for the ion selectivity are E280/E554/K1115/E1389 (EEKE), distinct from DEKA in Na_v_ channels and EEEE/EEDD in Ca_v_ channels. Sequence alignment of the P1-SF-P2 among NALCN, Ca_v_1.1, and Na_v_1.4 reveals an invariant tryptophan in the first residue of P2 and a highly conserved residue (Thr/Ser/Cys) in the last residue of P1, while the other residues are not very conserved (Fig. 5b). The overall structure of P1-SF-P2 in NALCN is more closely related to that of Ca_v_1.1 than Na_v_1.4, with r.m.s.d. values of 0.848 Å over 110 Cα atoms and 1.602 Å over 107 Cα atoms, respectively. The backbone of the pore helices of NALCN aligns well to those of Ca_v_1.1, whereas the P2s do not align well to those of Na_v_1.4 (Fig. 5c). Despite its structural similarity with Ca_v_ channels, NALCN is responsible for the background sodium leak conductance in hippocampal neurons, indicating that NALCN is selective for Na^+^ in vivo^4^.

To better understand Na^+^ binding and selectivity in SF, we performed canonical MD simulation to investigate the interaction of the membrane-embedded pore domain in 150 mM NaCl. In three independent trials of 200 ns equilibration, we observed three potential Na^+^ ion-binding sites, designated Site1-3, in proximity to SF (Fig. 5d). In addition to the glutamic acid residues in the SF, the Na^+^ binding sites are formed by several negatively charged residues from the pore helices, including E275, D558, D561, and D1390. Among the three binding sites, Site1 is most stably located in the middle of SF, while the other two are transient and presumably cation attractants (Fig. 5d and Supplementary Movie 1). Probability statistics suggest that there is an average of two Na^+^ ions within the 4 Å range of the SF residues over time (Fig. 5e). Notably, Site 3 formed by E280/E554/D558 (EED site) is spatially close to the Na^+^ binding site formed by a DEE motif in Na_v_ channels^35^ (Fig. 5f). However, the sidechains of the EED motif in NALCN are not as closely spaced as those of the DEE site in Na_v_ channels and may not form a favorable Na^+^ binding site. On the other hand, Site1, the most occupied binding site, is spatially close to a putative Ca^2+^ binding site in Ca_v_1.1 and Ca_v_3.1 channels^36,43^ (Fig. 5f). The replacement of glutamic acid (E) or aspartic acid (D) with lysine (K) in repeat III from Ca_v_ channels to NALCN shifts the ion selectivity from Ca^2+^ to monovalent ions. Like Na_v_ channels, the lysine in NALCN favors permeation of Na^+^ but not Ca^2+^ through the SF. However, the EEKE SF may still preserve the ability to bind Ca^2+^, as the structure of SF in repeat I/II/IV is almost identical to that of Ca_v_ channels (Fig. 5c). In this sense, extracellular Ca^2+^ may compete with Na^+^ in binding to SF, suggesting a potential mechanism of NALCN blockage by extracellular divalent cations^12^.

### Closed intracellular gate of NALCN

**Fig. 6:**
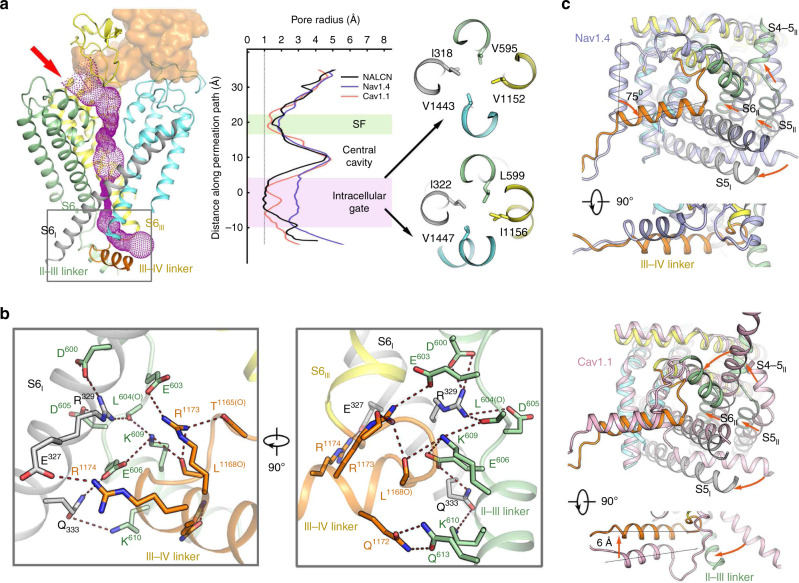
A closed intracellular pore. **a** Ion permeation path of NALCN. The pore region of NALCN is shown in cartoon and FAM155A is shown in the surface. The ion permeation path calculated by HOLE^46^ is illustrated by purple dots on the left. The extracellular ion entrance is indicated by a red arrow. The details in the rectangular region are shown in **b**. The corresponding pore radii along the permeation path of human NALCN (black), Na_v_1.4 (slate), and Ca_v_1.1(pink) are compared in the middle. Two narrowest layers of the intracellular gate are presented in extracellular views on the right. **b** Zoomed views of the intracellular gate. The electrostatic interactions are indicated by red dashed lines. The residues from S6_I_, II-III linker, and III-IV linker are colored black, green, and orange, respectively. **c** Pore structure comparisons between NALCN and Na_v_1.4 (top) or Ca_v_1.1 (bottom). NALCN is domain colored, and Na_v_1.4 and Ca_v_1.1 are colored in slate and light pink, respectively. An intracellular view and a side view are shown for each comparison. The major conformational changes from Na_v_1.4 or Ca_v_1.1 to NALCN are indicated by red arrows.

The ion permeation path below the SF in NALCN is enclosed by the S6 tetrahelical bundle, a structural feature that is shared in all reported Na_v_/Ca_v_ structures (Figs. 5a and 6a). Notably, a short seven residue π helix is embedded within each S6 segment (Supplementary Fig. 11a, b). The π helices are stabilized by intra-helical hydrogen bonds. Moreover, W311/L588/M1145/Y1436 of the π helix form close inter-helical contacts with L1439/K314/L591/L1148 from the adjacent π helix, sealing the lateral pore fenestration in NALCN (Supplementary Fig. 11b). We used HOLE^46^ to calculate the radius along the permeation path and compared it with that of Na_v_1.4 and Ca_v_1.1, whose intracellular gates are in open and closed states, respectively. Due to the existence of FAM155A, ions can enter the permeation path from only one side, which is above the pore domain of repeat II (Fig. 6a, left). Surprisingly, although NALCN is supposed to conduct a “leak” current, the intracellular gate is closed, even more tightly than the closed Ca_v_1.1. The narrowest region, sealed by two layers of the hydrophobic residues Val, Ile, and Leu, is ~10 Å in length along the permeation path with a radius <1 Å (Fig. 6a, right). Notably, the lower gate is fully blocked by the III-IV linker. Detailed analysis reveals that the III-IV linker is stabilized by extensive hydrogen bonds or polar interactions with S6_I_ and the II-III linker (Fig. 6b). Most of the involved residues are not conserved among Na_v_/Ca_v_ channels, indicating that the local interactions among S6_I_, the II-III linker and the III-IV linker are highly specific to NALCN, which implies that NALCN has a distinct gating mechanism (Supplementary Fig. 7).

It is intriguing that the position of the III-IV linker in NALCN is distinct from those of both Na_v_1.4 and Ca_v_1.1 in different ways. NALCN does not have an IFM motif, a highly conserved motif in the III-IV linker of all Na_v_ channels that is crucial for fast inactivation through an allosteric inhibition mechanism^45,47^. In Na_v_1.4, the III-IV linker is away from the inner pore, and its helix rotates counterclockwise by ~75° but keeps at a similar vertical height to that of NALCN (Fig. 6c, top). The helix in the III-IV linker, however, shows a similar orientation but distinct vertical height between NALCN and Ca_v_1.1. Compared to Ca_v_1.1, the helix in the III-IV linker of NALCN is shifted ~6 Å up towards the extracellular side, probably due to a shorter loop connecting S6_III_ and the helix in the III-IV linker (Fig. 6c, bottom). The II-III linker, which is part of the elongated S6_II_ in Ca_v_1.1, bends towards the center pore. These distinct structural features in NALCN make the S6_I_, II-III linker and III-IV linker forms a close contact that tightly seals the inner gate.

Like Ca_v_1.1 and Na_v_PaS, the III-IV linker of NALCN also interacts with the CTD (Fig. 1c). The CTD of NALCN consists of at least six short helices C1-C6, among which C1-C4 form an EF-hand-like domain that is structurally conserved in the CTD of Ca_v_1.1, Na_v_PaS, and Na_v_1.5. However, C5-C6 helices in NALCN are distinct from other channels (Supplementary Fig. 12). The conserved IQ motif which is a binding site for calmodulin in the C6 helix of Na_v_ channels is absent in NALCN. Interestingly, when the CTDs are superimposed, the III-IV linker of NALCN displays a shift compared to that of Ca_v_1.1 and Na_v_PaS, or coincides with the C5 helix of Na_v_1.5, suggesting that the CTD and the III-IV linker may undergo relative conformational changes (Supplementary Fig. 12). Speculatively, CTD may provide specialized binding sites for UNC80 or UNC79 in the regulation of the gating of NALCN.

### A unique lipid-binding site on NALCN

Similar to S4-5_I_ and S5_I_, the S4-5_II_, S5_II_, and S6_II_ segments in NALCN also displays significant structural deviations compared to that of Ca_v_1.1/Na_v_1.4 (Fig. 6c). The dramatic differences in the pore domain of repeat II may be related to the binding of a lipid molecule (lipid1) in a nearby cavity (Fig. 2c). We have assigned a total of ten lipid molecules in the structure. Most of the lipids in the structure bind at semi-closed clefts around the protein, while only lipid1 sits in a closed hydrophobic cavity surrounded by S4-5_I_, S5_I_, S6_I_, S5_II_, and S6_II_ (Fig. 1c, Supplementary Fig. 13a, b). Although the exact identity of lipid1 is not clear, the two-tail shape density suggests it may be a phospholipid (Supplementary Fig. 4b). Two positively charged residues Lys504 and Lys505 from S5_II_ can form hydrogen bonds with the phosphate group of lipid1, providing the molecular basis of binding specificity. Notably, such lipid binding is not observed in other channels, implying an important role in regulating the function of NALCN. Indeed, mutations on the two positively charged residues, such as K504A, K504D, K505A, result in a significant reduction in the measured currents conducted by NALCN (Supplementary Fig. 13c). In contrast, mutations on the hydrophobic residues along the lipid-binding cavity, such as F325A, I328W, increase the measured current, while F332A has little effect on the current. These results suggest that except for the auxiliary subunits, the function of NALCN is also fine-tuned by specific lipid–protein interaction.

## Discussion

Our study reports the high-resolution structure of NALCN in complex with the auxiliary subunit FAM155A. The structure reveals unique features of NALCN that help explain its ion selectivity, voltage sensing, and specific interaction with auxiliary subunits. It also provides an important framework for comparative investigations of the channel properties of related Na_v_/Ca_v_ channels.

The sample for structural investigation in this study was prepared in detergent micelle and the cryo-EM map and structure turns out to be very similar to that of the sample prepared in nanodisc^31^ (Supplementary Fig. 14). Although the resolution is slightly lower, the map in detergent micelle shows higher quality in the extracellular region than the map in nanodisc (Supplementary Fig. 14b). When the maps are contoured at low levels, the size of the detergent micelle is slightly larger than that of nanodisc. Some extra densities that may belong to the unresolved region of FAM155A and CTD can be observed in both maps (Supplementary Fig. 14a). Even though UNC79 and UNC80 were coexpressed in the samples, no obvious density for UNC79 and UNC80 was observed in both maps.

**Fig. 7:**
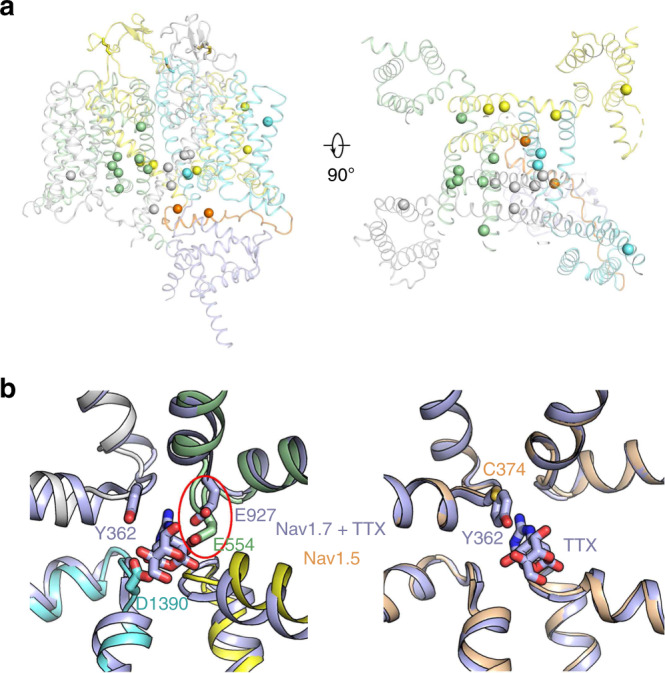
Disease mutations mapping on NALCN and TTX incompatibility of NALCN. **a** Please also refer to Supplementary Table 3 for a summary of the disease-related mutations on human NALCN. The Cα atoms of the residues whose mutations lead to human diseases are mapped to the structure of NALCN and shown as spheres. The mapping is shown on both sides and extracellular views. **b** Superimposition of the pore helices and SF of NALCN with that of TTX-bound Na_v_1.7 (left). The determinant Tyr362 for TTX coordination in Na_v_1.7 is not conserved in NALCN and the structural deviations in the pore region of NALCN also result in potential clashes with the TTX binding site, making NALCN and TTX incompatible. As a comparison, despite lack of the determinant Tyr, TTX-insensitive Na_v_1.5 shows a similar backbone structure of pore helices and SF to that of TTX-bound Na_v_1.7, thus retains a lower binding affinity to TTX (right).

Unlike Na_v_/Ca_v_ channels, there is only one subtype of NALCN in most organisms including humans. Certain mutations/deletions of NALCN can be fatal as there are no channels with redundant functions. NALCN is essential for neonatal survival and NALCN knockout mice die within 24 h after birth^4^. To investigate the molecular mechanisms of NALCN channelopathies, we summarized the reported disease-related mutations and mapped them onto the structure (Fig. 7a and Supplementary Table 3). We found that most of the mutations are located on the pore domain of NALCN (Fig. 7a). These mutations may affect NALCN function by directly altering ion permeation properties through the pore. Two mutations on the III-IV linker, T1165P and R1181Q, may lead to changes in channel gating by affecting the local interactions of the III-IV linker with adjacent S6_I_, the II-III linker, S6_IV_ and the CTD. Our structure thus provides an important framework to interpret the potential disease-causing mechanisms of the reported mutations.

TTX is a neurotoxin and sodium channel blocker. The Na^+^ current conducted by NALCN is TTX-resistant^4^. Our structure directly conveys the incompatibility of TTX with NALCN pores. Previous structural comparison between TTX-bound Na_v_1.7 and apo Na_v_1.5 revealed that a residue with an aromatic ring (Tyr362 in Na_v_1.7) in repeat I is the structural determinant of TTX sensitivity in Na_v_ channels^39^. This residue forms a π–π stacking interaction with TTX-bound in the outer pore of Na_v_1.7, and replacement of the residue with cysteine in TTX-insensitive Na_v_1.5 results in much lower TTX affinity (Fig. 7b). In NALCN, not only is the determinant residue replaced by glycine, but the other key residues involved in TTX binding are also not conserved (Supplementary Fig. 8). Moreover, the overall backbone of the pore helices in NALCN has changed considerably compared with Na_v_1.7, which led to clashes within the potential TTX binding site (Fig. 7b). These structural features of NALCN make it unable to bind TTX and even more resistant to TTX than TTX-insensitive Na_v_ channels.

The auxiliary subunits have important roles in fine-tuning the activity of NALCN. FAM155A, previously characterized as an ER-resident protein in *C. elegans*^11^, turns out to interact directly with NALCN in humans. It may help NALCN fold properly and translocate to the cell membrane. Our observation of a clear left shift of the G–V curve upon additional UNC79 and UNC80 coexpression with NALCN and FAM155A, suggested an additional regulatory effect of UNC79 and UNC80 on NALCN (Supplementary Fig. 1). NALCN contains a π helix in each S6 segment (Fig. 5a and Supplementary Fig. 11b). Conformational changes of the channel through the transitions between π helices and α helices of the S6 segments have been reported in many structures^36,48,49^. For instance, the opening of TRPV6 is coupled with α-to-π transitions in the S6 segments^48^ (Supplementary Fig. 11c). It is likely that NALCN may also open the gate through similar π-to-α helical transitions, which could be triggered by many factors, including the association of UNC79 and UNC80. Additional components, such as several GPCRs and SFKs, may also contribute to the modulation of NALCN function. It remains an open question whether there are additional auxiliary subunits of the NALCN channelosome. Future structural and functional studies on larger complexes of NALCN will deepen our understanding of the regulation of the NALCN channelosome.

## Methods

### Protein expression and purification

NALCN, UNC-80, UNC-79, and FAM155A were cloned into pCAG vector. HEK 293F cells (Invitrogen) were transfected with the four plasmids and harvested after 60 h. Cells were resuspended in buffer containing 25 mM Mops (pH 7.4), 250 mM NaCl, 0.5% (w/v) Lauryl Maltose Neopentyl Glycol (LMNG, Anatrace), 0.06% (w/v) cholesteryl hemisuccinate Tris salt (CHS, Anatrace) and protease inhibitor cocktail including 2 mM phenylmethylsulfonyl fluoride (PMSF), 1.3 μg/ml aprotinin, 0.7 μg/ml pepstatin, and 5 μg/ml leupeptin, then incubated at 4 °C for 2 h. The insoluble fraction was precipitated by ultracentrifugation at 255,700×*g* for 1 h, and the supernatant was applied to anti-Flag G1 affinity resin (GenScript) by gravity. The resin was rinsed several times with the Wash buffer, which contains 25 mM Mops (pH 7.4), 500 mM NaCl, 0.01% (w/v) Lauryl Maltose Neopentyl Glycol, and the protease inhibitor cocktail. The target proteins were eluted by the Elution buffer containing 25 mM Mops (pH 7.4), 500 mM NaCl., 0.02% (w/v) glyco diosgenin (GDN, Anatrace), and the protease inhibitor cocktail plus 200 μg/ml FLAG peptide (GenScript). The eluent was then concentrated using a 50-kDa cut-off Centricon (Millipore) and further purified by Superose 6 Increase 10/300 GL column (GE Healthcare) in 25 mM Mops (pH 7.4) 150 mM NaCl, 0.02% (w/v) GDN, and the protease inhibitor cocktail. Fractions were collected and concentrated to about 8.5 mg/ml for cryo-EM analysis.

### Whole-cell electrophysiology

Patch-clamp recordings were performed with a HEKA EPC10 amplifier with PatchMaster software (HEKA) in whole-cell configuration. Patch pipettes were prepared from borosilicate glass and fire-polished to resistance of ~6 MΩ. For whole-cell recording, serial resistance was compensated by at least 65%. Extracellular solution contained 150 mM NaCl, 10 mM Hepes, and 30 mM d-(+)-glucose (pH 7.4) with NaOH, and intracellular solution contained 136 mM NaCl, 5 mM EGTA, 10 mM Hepes, and 2 mM Na_2_ATP (adenosine 5′-triphosphate) (pH 7.2) with NaOH. The current amplitude at the steady-state during the last 20 ms of voltage steps was averaged to construct the G–V curve. All recordings were performed under room temperature (~22 °C). Temperature variation was less than 1 °C as monitored by a thermometer. Current signal was sampled at 20 kHz.

HEK-293T cells were grown in Dulbecco’s modified Eagle’s medium (ThermoFisher Scientific) supplemented with 10% fetal bovine serum and 1% penicillin-streptomycin at 37 °C in a 5% CO2 humidified growth incubator. Cells were transiently transfected by Lipofectamine 3000 (Life Technologies) 48–72 h before patch-clamp recordings. The NALCN, UNC-79, UNC-80, and FAM155A cDNAs were mixed in the mass ratio of 1:1:1:1.

To apply solutions containing ligands such as verapamil during patch-clamp recording, a rapid solution changer with a gravity-driven perfusion system was used (RSC-200, Bio-Logic). Each solution was delivered through a separate tube so that there was no mixing of solutions. Pipette tip with a membrane patch was placed directly in front of the perfusion outlet during recording. Each membrane patch was recorded only once.

Data from patch-clamp recordings were analyzed in Igor Pro (WaveMatrix). To characterize the steady-state G–V curves, a single-Boltzmann function was used:

1 $$\frac{{{G}}}{{{{G}}_{{\mathrm{max}}}}} = \frac{1}{{1 + {{e}}^{ - \frac{{q_{app} \cdot {{F}}}}{{{{RT}}}}\left( {{{V}} - {{V}}_{1/2}} \right)}}},$$

where *G*/*G*_max_ is the normalized conductance, *V*_1/2_ is the half-activation voltage, *q*_app_ is the apparent gating charge and *F* is Faraday’s constant, *R* is gas constant, and *T* is the temperature in Kelvin, which is set to 295 K (22 °C).

### Cryo-EM sample preparation and data collection

The purified NALCN complex was concentrated to about 8.5 mg/ml for cryo-sample preparation. Aliquots (3.5 μl) of protein solution were loaded onto glow-discharged holey carbon grids (Quantifoil Au R1.2/1.3), which were blotted for 3.5 s and immersed in liquid ethane cooled by liquid nitrogen using Vitrobot (Mark IV, ThermoFisher Scientific). The grids were exposed through Titan Krios operating at 300 kV equipped with Gatan K3 Summit detector and GIF Quantum energy filter in super-resolution mode (81,000× magnification). Movie stacks were automatically acquired using AutoEMation^50^, with a 20 eV slit width and a defocus range from −0.5 to −2.5 μm. Each stack consisting of 32 frames was exposed for 2.56 s with 0.08 s per frame, and for ~50 e^−^/Å^2^ of the total dose.

### Cryo-EM data processing

Collected movie stacks were motion-corrected by MotionCor2^51^ with 2-fold binning, producing micrographs of 1.087 Å pixel size. Following patch-CTF estimation, around eleven million particles were automatically picked with cryoSPARC v2^52^ from 17,922 micrographs. Three rounds of 2D classification enriched images of good classes, resulting in a total of 905,458 particles being selected. An Ab-initio reconstruction generated a low-resolution initial map for the subsequent homogeneous and non-uniform refinement jobs^53^, yielding a 3.8-Å reconstruction with identifiable side-chain densities in NALCN pore domain only. To improve map quality, we exported the aligned particles and did a 3D classification without orientation assignment using RELION 3.0 (--skip-align flag)^54^. One out of five 3D classes represented clear secondary structures of the overall channel complex, and the 65,177 particles were fed back to cryoSPARC v2 for further homogeneous and non-uniform refinements that pushed the resolution to 3.14 Å. Adapted masks for protein regions, extracellular subunit, and intracellular domain were applied in particle subtraction and local refinement tasks, which helped in achieving slightly higher resolution at 3.11, 2.94, and 3.04 Å, respectively. Map resolutions were determined by the gold-standard Fourier shell correlation (FSC) 0.143 criterion using Phenix.mtriage^55^.

### Model building and refinement

An initial model of NALCN was generated using SWISS-MODEL online server^56^. The template used for the homology modeling was the α1 subunit of Ca_v_1.1 (PDB: 5GJV). The model was firstly docked into the final reconstruction map in Chimera^57^ and then manually adjusted in COOT^58^. After building the model of NALCN, an extra density remained unmodelled, which is close to the ECL of NALCN in the extracellular side. The high quality of the map enables us to identify that the extra density belongs to FAM155A. The structure of FAM155A was then de novo built. The sequence assignment was guided by bulky residues such as Phe, Tyr, and Trp. Up to six disulfide bonds and a glycosylation site (Asn217) were identified in FAM155A, further verifying the accuracy of the model. Ten lipid molecules were manually built to fit into the corresponding densities. The density for the C6 helix of CTD is discontinuous and has been tentatively assigned as NALCN (1589-1602) based on the side chain density.

Subsequently, the models were refined against the corresponding maps by PHENIX^55^ in real space (phenix.real_space_refine) with secondary structure and geometry restraints generated by ProSMART^59^. Overfitting of the overall models was monitored by refining the models in one of the two independent half maps from the gold-standard refinement approach and testing the refined model against the other map^60^. Statistics of 3D reconstruction and model refinement can be found in Supplementary Table 2.

### Molecular dynamics simulation

The pore domain of NALCN (residue 171–204, 260–328, 490–603, 1001–1042, 1094–1163, 1318–1400, and 1420–1455) was embedded in 1-palmitoyl-2-oleoyl-sn-glycero-3-phosphocholine (POPC) bilayer for molecular dynamics simulations. After the determination of side-chain protonation states by PROPKA3.1^61^ and peptide terminal neutralization by acetyl/methylamine capping groups, water molecules and 150 mM NaCl were added using VMD^62^, resulting in a system of size 100 × 100 × 105 Å with ~93,000 atoms. The system was parameterized using tleap in AmberTools18^63^ by ff14SB^64^ and LIPID17 force fields, and along with TIP3P water model^65^. All the simulations were performed using OpenMM7^66^. We first applied positional constraints (*k* = 10 kcal/mol/Å^2^) on protein-heavy atoms for a 5000-step minimization, then released constraints of side chains when heating up the system to 310 K with 2 fs stepsize (H-bonds constraints), 1 ps^−1^ friction coefficient for Langevin dynamics, Particle Mesh Ewald (PME) method^67^, and 12 Å non-bonded cut-off. During 50-ns pre-equilibration for membrane relaxation in NPT ensemble, the force constant k of backbone atoms was reduced from 10 to 0.1 kcal/mol/Å^2^. Three independent 200-ns production runs were conducted constraining Cα atoms more than 15 Å away from the selectivity filter residues (EEKE) with *k* = 0.1 kcal/mol/Å^2^. Simulation trajectories were analyzed using MDTraj 1.9.3^68^. After superimposition of the pore region to the initial structure, we recorded the positions of sodium ions within 15 Å of SF residues EEKE every 1 ns for each run.

### Protein crosslinking and LC–MS/MS analysis

Equal amount (w/w) of BS3 (bis[sulfosuccinimidyl] suberate) was added to the protein mixture, which was incubated at room temperature for 1 h. The reaction was terminated by adding 0.5 M ammonium bicarbonate to a final concentration of 20 mM for 10 min incubation. The SDS-PAGE was used to separate the crosslinked protein and stained with Coomassie Blue G-250. The gel bands of interest were cut into pieces. Sample was digested by trypsin with prior reduction and alkylation in 50 mM ammonium bicarbonate at 37 °C overnight. The digested products were extracted twice with 1% formic acid in 50% acetonitrile aqueous solution and dried to reduce volume by speedvac.

For LC–MS/MS analysis, the peptides were separated by a 65 min gradient elution at a flow rate 0.300 µl/min with the Thermo EASY-nLC1200 integrated nano-HPLC system which is directly interfaced with the Thermo Q Exactive HF-X mass spectrometer. The analytical column was a home-made fused silica capillary column (75 µm ID, 150 mm length; Upchurch, Oak Harbor, WA) packed with C-18 resin (300 A, 3 µm, Varian, Lexington, MA). Mobile phase A consisted of 0.1% formic acid, and mobile phase B consisted of 100% acetonitrile and 0.1% formic acid. The mass spectrometer was operated in the data-dependent acquisition mode using the Xcalibur 4.1 software and there is a single full-scan mass spectrum in the Orbitrap (300–1800 *m*/*z*, 60,000 resolution) followed by 20 data-dependent MS/MS scans at 30% normalized collision energy. Each mass spectrum was analyzed using the Thermo Xcalibur Qual Browser and pLink 2^69^ for the database searching and cross-linking analysis.

### Reporting summary

Further information on research design is available in the Nature Research Reporting Summary linked to this article.

## Supplementary information

Supplementary Information

Reporting Summary

Description of Additional Supplementary Files

Supplementary Movie 1

## Data Availability

Data supporting the findings of this manuscript are available from the corresponding authors upon reasonable request. Atomic coordinate and corresponding EM maps of the NALCN-FAM155A complex PDB 7CM3 and EMD-30400 have been deposited in the Protein Data Bank (http://www.rcsb.org) and the Electron Microscopy Data Bank (https://www.ebi.ac.uk/pdbe/emdb/), respectively. The crosslinking mass spectrometry data have been deposited in the PRIDE database (https://www.ebi.ac.uk/pride/) with the accession number of ‘PXD022119’. Source data are provided with this paper.

## Source data

Source Data
