# Supplementary material for: Structure of the human sodium leak channel NALCN in complex with FAM155A
Source: Nat Commun. 2020 Nov 17;11:5831. doi: 10.1038/s41467-020-19667-z (PMC7672056; doi:10.1038/s41467-020-19667-z)
Supplement: Supplementary file 1 — Supplementary Information [file 41467_2020_19667_MOESM1_ESM.pdf]

Supplementary Information for

Structure of the human sodium leak channel NALCN in complex with FAM155A

Jiongfang Xie, Meng Ke, Lizhen Xu, Shiyi Lin, Jin Huang, Jiabei Zhang, Fan Yang*, Jianping Wu*, and Zhen Yan*

*To whom correspondence should be addressed: Z. Yan (yanzhen@westlake.edu.cn), J. Wu (wujianping@westlake.edu.cn), or F. Yang (fanyanga@zju.edu.cn)

This file includes:

Supplementary Tables 1-3
Supplementary Figs. 1-14

Supplementary Table 1. Data collection and model statistics

**Data collection**	
EM equipment	Titan Krios
Voltage (kV)	300
Detector	Gatan K3
Energy filter	Gatan GIF Quantum, 20 eV slit
Pixel size (Å)	1.087
Electron dose (e^-/Å^2)	50
Defocus range (μm)	-0.5~-2.5
Data set	NALCN-FAM155A complex
Number of images	17,922
**Reconstruction**	
Software	RELION 3.0 / CryoSPARC v2
Initial number of particles	10,763,738
Number of used Particles	65,177
Symmetry	C1
Final Resolution (Å)	3.1
Map resolution FSC threshold	0.143
Map resolution range	2.8-4.0
Map sharpening B-factor (Å^2)	-86.5
**Model building and refinement**	
Model building software	Coot
Refinement software	Phenix
Initial model	Ab Initio reconstruction
Model resolution FSC threshold	0.5
**Model composition**	
Protein residues	1,460
Side chains	1,460
Sugar	7
Lipid	10
**Validation**	
Molprobity score	1.63
Clash score	6.35
Poor rotamers (%)	0.08
R.m.s deviations	
Bonds length (Å)	0.006
Bonds Angle (°)	0.744
Ramachandran plot statistics (%)	
Preferred	96.0
Allowed	4.0
Outlier	0.0

Supplementary Table 2. Summary of contact interfaces between NALCN and FAM155A

NALCN	Residue	FAM155A	Residue
ECL_{III}	W1085 (O)	H5	Y308
ECL_{III}	W1085	H3	E281
ECL_{III}	R1062	H3	E281
ECL_{I}	I224 (N)	H3	K288 (O)
ECL_{III}	R1054	L2	E366
ECL_{III}	W1090 (O)	L2	L364 (N)
ECL_{III}	D1099	L2	Y365
ECL_{III}	N1047 (O)	L2	Y353
ECL_{III}	N1064 (O)	L2	D347 (N)
ECL_{III}	K1069	L2	D347
ECL_{III}	K1069	L2	N348 (O)
ECL_{III}	NAG (O3)	L2	E350
$P2_{III}$	R1127	L2	I352 (O)
ECL_{III}	R1094 (N)	L2	G354 (O)
ECL_{IV}	P1403 (O)	H6	T339
ECL_{III}	D1048	L1	K298
ECL_{IV}	A1414 (O)	L1	K298
ECL_{IV}	D1416	L1	S296
ECL_{IV}	Y1362 (N)	L1	Y295 (O)
ECL_{IV}	R1368	L1	Q291
ECL_{IV}	K1361	H6	C341 (O)

ECL: Extracellular loop

Supplementary Table 3. Disease related mutations on human NALCN

Protein	Mutations	Diseases	Structural Mapping	Reference
hNALCN	R43Q	EBD	$S1_{I}$	(*1*)
hNALCN	W107*	IHPRF1	$S3_{I}$	(*2*)
hNALCN	Q177P	CLIFAHDD	$S5_{I}$	(*3*)
hNALCN	W179*	IHPRF1	*Gene shift*	(*2*)
hNALCN	L312I	CLIFAHDD	$S6_{I}$	(*3*)
hNALCN	L312V	NDH	$S6_{I}$	(*1*)
hNALCN	V313G	CLIFAHDD	$S6_{I}$	(*3*)
hNALCN	F317C	DACH	$S6_{I}$	(*4*)
hNALCN	E327K	CLIFAHDD	$S6_{I}$	(*3*)
hNALCN	Y497T	ARSSHSICD	$S4\text{-}S5_{II}$	(*5*)
hNALCN	L509S	CLIFAHDD	$S5_{II}$	(*3*)
hNALCN	F512V	CLIFAHDD	$S5_{II}$	(*3*)
hNALCN	T513N	CLIFAHDD	$S5_{II}$	(*3*)
hNALCN	Y578S	CLIFAHDD	$S5_{II}$	(*3*)
hNALCN	L590F	CLIFAHDD	$S6_{II}$	(*3*)
hNALCN	V595F	DACH	$S6_{II}$	(*4*)
hNALCN	Q642*	INAD	II-III linker	(*6*)
hNALCN	C675L	IHPRF1	II-III linker	(*7*)
hNALCN	E813G	IHPRF1	II-III linker	(*2*)
hNALCN	Q877N	IHPRF1	$S0\text{-}S1_{III}$	(*2*)
hNALCN	V891S	IHPRF1	$S1_{III}$	(*2*)
hNALCN	I920L	IHPRF1	$S2_{III}$	(*2*)
hNALCN	V956-L963del	IHPRF1	$S3_{III}$	(*2*)
hNALCN	V1006A	CLIFAHDD	$S4\text{-}S5_{III}$	(*3*)
hNALCN	R1008W	IHPRF1	$S4\text{-}S5_{III}$	(*2*)
hNALCN	I1017T	CLIFAHDD	$S5_{III}$	(*3*)
hNALCN	L1019F	IHPRF1	$S5_{III}$	(*2*)
hNALCN	V1020F	NDH	$S5_{III}$	(*1*)
hNALCN	T1165P	CLIFAHDD	III-IV linker	(*3*)
hNALCN	R1181Q	NDH	III-IV linker	(*1*)
hNALCN	Q1186*	IHPRF1	III-IV linker	(*2*)
hNALCN	W1287L	IHPRF1	$S3_{IV}$	(*5*)
hNALCN	R1304X	PB	$S4_{IV}$	(*7*)
hNALCN	R1384*	IHPRF1	$P1_{IV}$	(*2*)
hNALCN	F1427L	IHPRF1	$S6_{IV}$	(*2*)
hNALCN	I1445L	IHPRF1	$S6_{IV}$	(*2*)
hNALCN	I1446M	CLIFAHDD	$S6_{IV}$	(*3*)
hNALCN	*1489ΔT*	IHPRF1	*Gene shift*	(*5*)

Note：* represents premature translational termination

CLIFAHDD: Congenital contractures of the limbs and face, hypotonia, and developmental delay; **IHPRF1**: Hypotonia, infantile, with psychomotor retardation and characteristic facies 1; **EBD**: Exaggerated body dends; ***DACH***: Distal arthrogryposis and central hypertonia; ***NDH***: Neurodevelopmental Disease and Hypotonia; ***PB***: Periodic breathing; ***ARSSHSICD***: Autosomal recessive syndrome with severe hypotonia, speech impairment, and cognitive delay; **INAD**: Infantile neuroaxonal dystrophy

Supplementary Figures and Legends

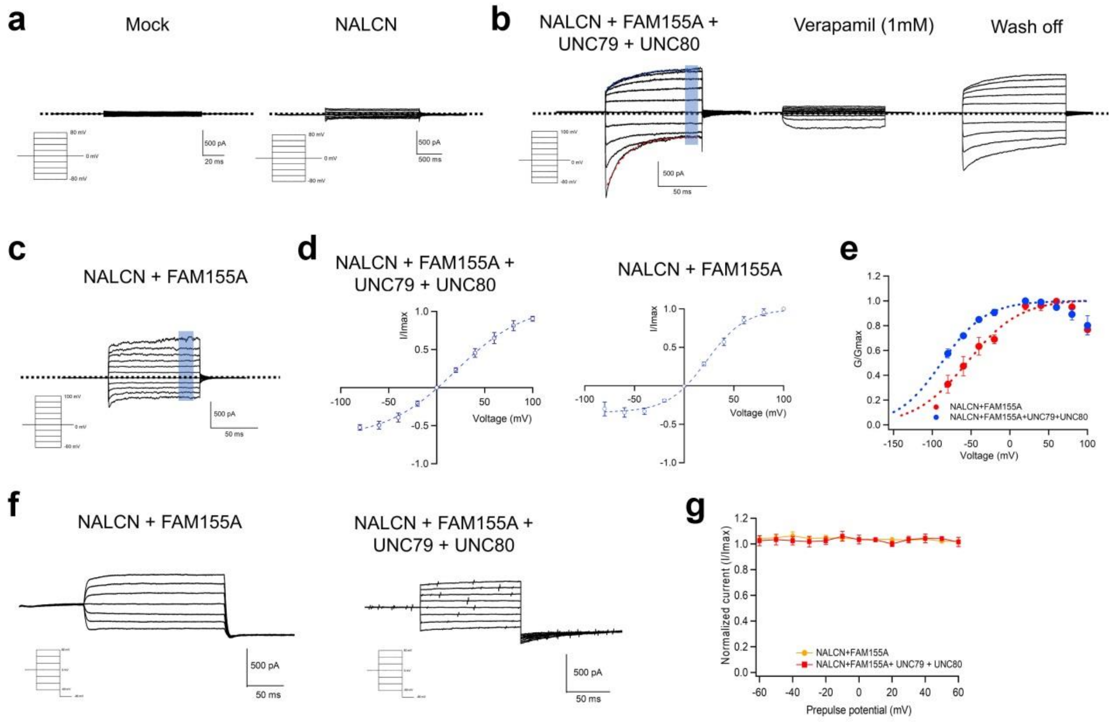

Supplementary Fig. 1. Electrophysiological properties of NALCN characterized by patch-clamp recordings. **(a)** Representative whole-cell recordings of HEK293 with mock (empty vector) and NALCN transfected, respectively. Dashed line indicates the zero-current level. **(b)** Representative whole-cell recordings of NALCN, FAM155A, UNC79 and UNC80 co-expressed in HEK293 cells. Data points in shaded blue rectangle were averaged for plotting current-voltage curve in **(d)**. Dashed curves in red and blue are fitted curves to the inactivation and activation kinetics with exponential functions, respectively. NALCN current was inhibited by 1 mM verapamil and the current could be recovered after washing off. The same whole-cell patch was used during verapamil inhibition. **(c)** Representative whole-cell recordings of NALCN and FAM155A co-expressed in HEK293 cells. Data points in shaded rectangle were averaged for plotting current-voltage curve. **(d)** Current-voltage curve of NALCN co-expressed with FAM155A only (n = 6) and co-expressed with FAM155A, UNC79 and UNC80 (n = 7). The currents were normalized to the maximum current level. **(e)** Conductance-voltage (G-V) curves of NALCN co-expressed with FAM155A (Red: $V_{1/2}$ = -44.66 ± 1.91 mV; apparent

gating charge (Z_{app}) = 1.22 ± 0.18 e_0) and co-expressed with FAM155A, UNC79 and UNC80 (Blue: $V_{1/2}$ = -65.07 ± 2.11 mV; Z_{app} = 1.25 ± 0.55 e_0). G-V curves were fitted to a single-Boltzmann function (equation (1) in Methods). Co-expression with FAM155A, UNC79 and UNC80 shifted the G-V curve of NALCN channel towards the hyperpolarization voltage. All data are given as mean ± s.e.m. **(f)** Representative whole-cell recordings of the steady-state inactivation of NALCN co-expressed with FAM155A (*left*) or with FAM155A, UNC79 and UNC80 (*right*). **(g)** Prepulse voltage dependence of steady-state inactivation of NALCN channel co-expressed with auxiliary subunits measured from -80 mV (n = 7). Source data are provided as a Source Data file.

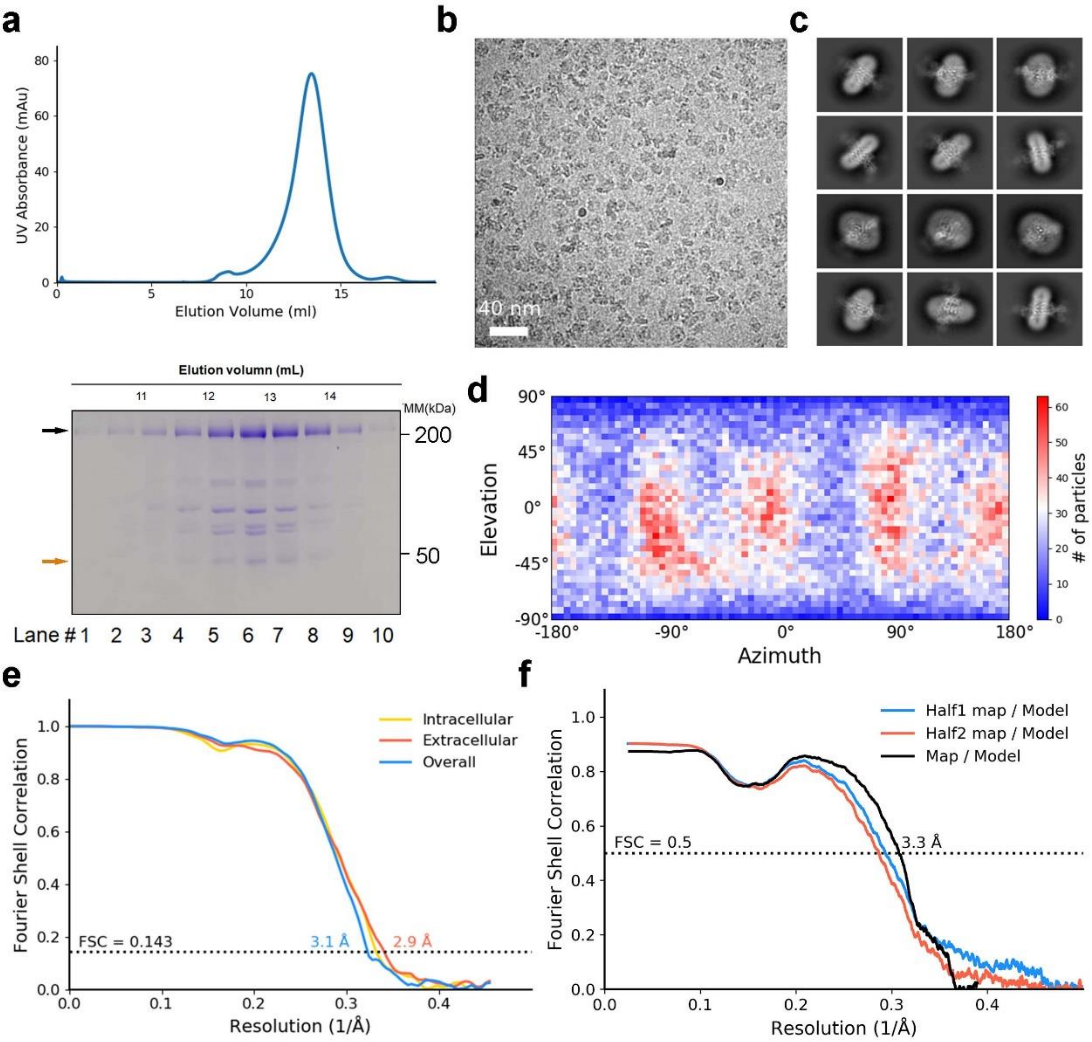

Supplementary Fig. 2. Cryo-EM analysis of human NALCN in complex with FAM155A. **(a)** Last step purification of recombinantly expressed NALCN-FAM155A complex. A representative chromatogram of gel filtration purification is shown. The indicated fractions were resolved by SDS-polyacrylamide gel electrophoresis followed by Coomassie blue staining. The bands corresponding to NALCN and FAM155A are indicated by black and orange arrows, respectively. The protein identity of each band was confirmed by mass spectrometric analysis. **(b)** Representative micrograph of the NALCN-FAM155A complex. Scale bar, 40 nm. **(c)** Representative two-dimensional class averages of NALCN-FAM155A complex. Box size: 240 Å; circle mask: 220 Å. **(d)** Angular distribution of the particles of the final reconstruction generated by cryoSPARC. **(e)** Gold standard FSC curves for the

3D reconstructions. The curves for the reconstructions of the overall map, map of the intracellular region and map of the extracellular region are indicated by blue, yellow, and red lines, respectively. **(f)** Validation of the final structure models. FSC curves of the final refined model versus the corresponding map that it was refined against (black); of the model refined in the first of the two independent half maps used for the gold-standard Fourier shell correlation curves versus that same map (blue); and of the model refined in the first of the two independent maps versus the second independent map (red). The small difference between the blue and red curves indicates that the refinement of the atomic coordinates did not suffer from overfitting.

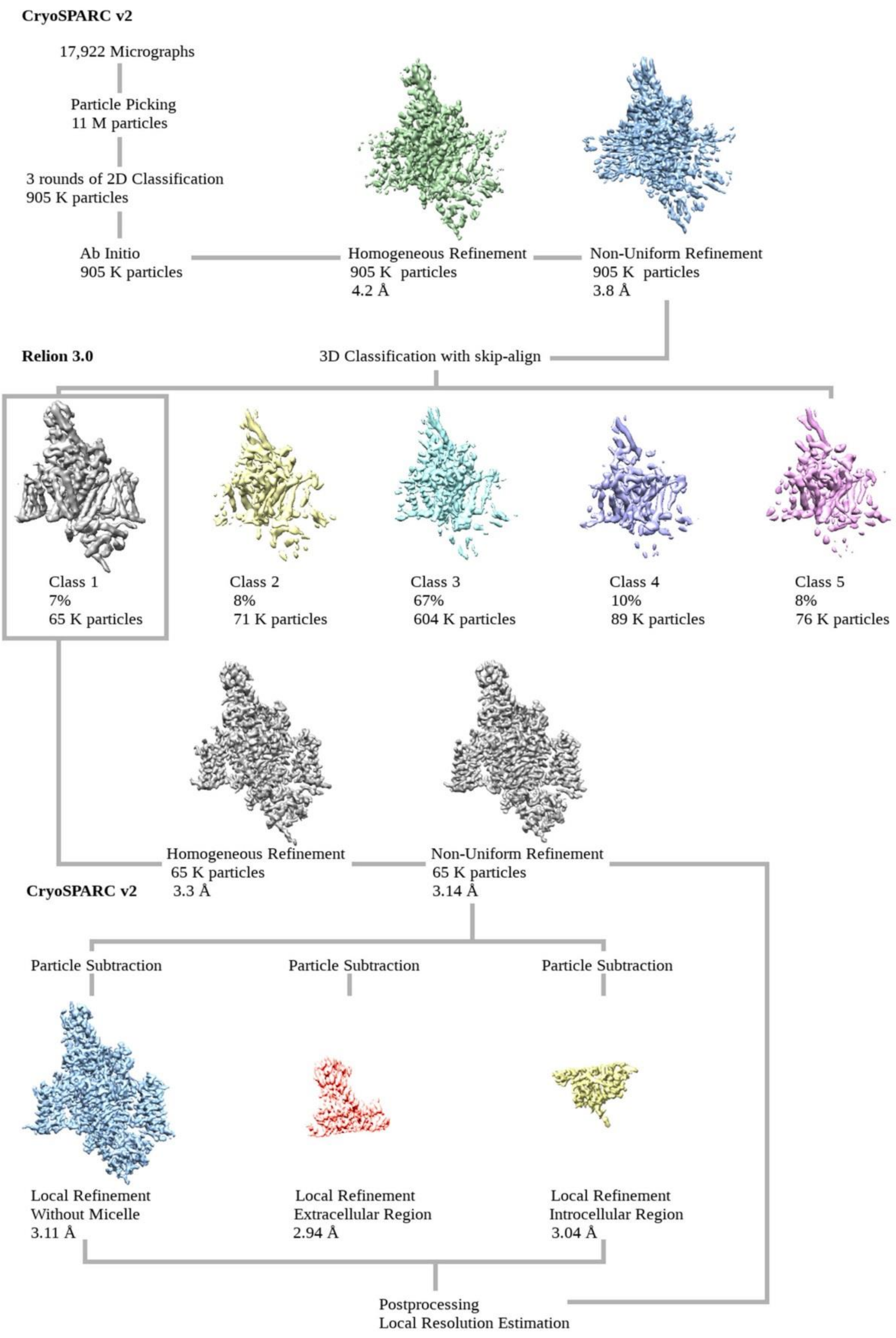

Supplementary Fig. 3. Flowchart for EM data processing. Please refer to "Image processing" session in Methods for details.

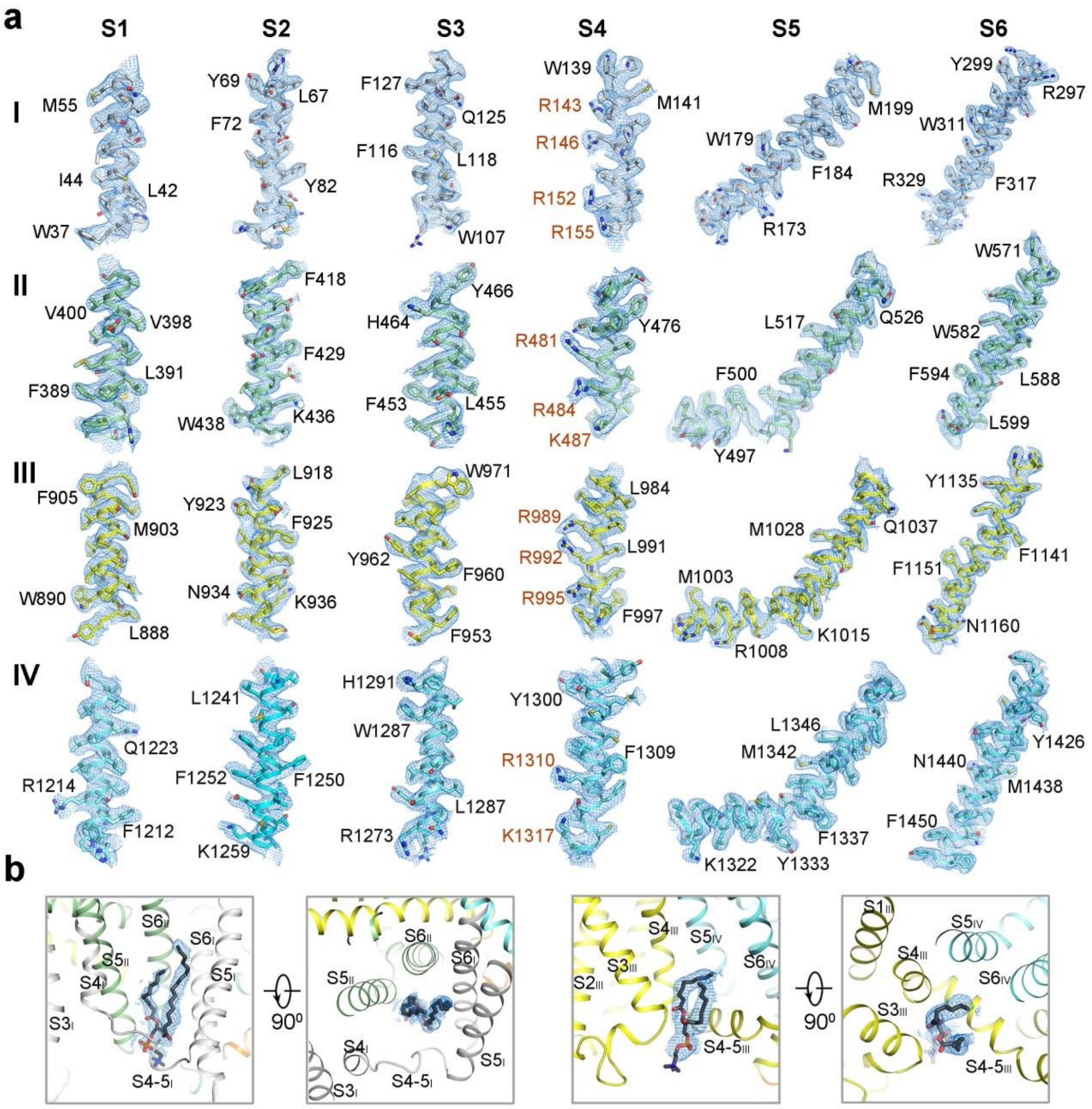

Supplementary Fig. 4. EM maps of human NALCN and lipids. (a) EM maps for the S1-S6 segments in the four repeats of NALCN. The side groups of representative bulky residues are labeled. The gating charges in S4 helices are highlighted in brown. The densities, shown as blue meshes, are contoured at 4-5 σ in PyMol. **(b)** EM maps of two representative lipid molecules. Two perpendicular views are shown for each lipid. The lipid densities are contoured at 3-4 σ in PyMol.

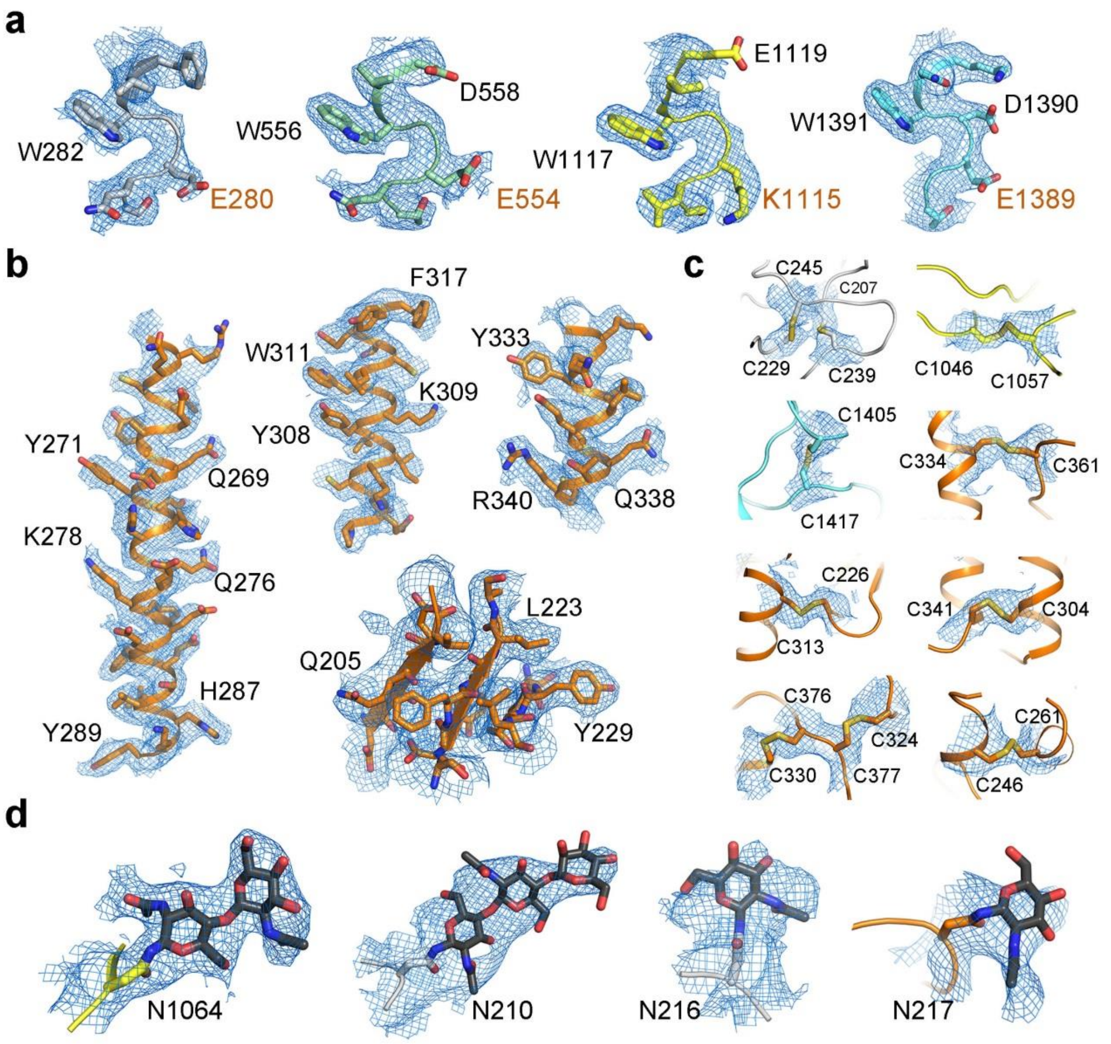

Supplementary Fig. 5. EM maps of NALCN-FAM155A complex. (a) All residues that constitute the SF in four repeats of NALCN are clearly resolved. The densities are contoured at 4-5 σ in PyMol. The EEKE residues are labeled orange. **(b)** Representative EM maps of FAM155A. The side groups of bulky residues are labeled. **(c)** EM maps of the disulfide bonds in the complex structure. The densities are contoured at 3-4 σ in PyMol. **(d)** EM maps of the sugar moieties in the glycosylation sites. The densities are contoured at 2-4 σ in PyMol.

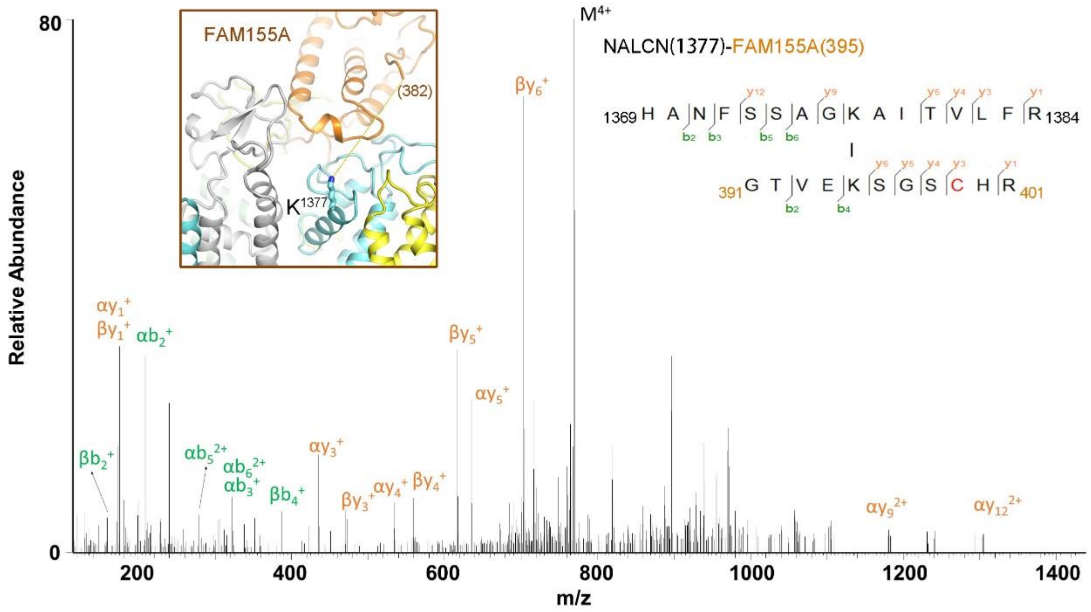

Supplementary Fig. 6. Mass Spectrometric analysis of crosslinked NALCN-FAM155A complex. Shown here is a high resolution HCD spectra of the inter-subunit crosslinked peptides from NALCN-FAM155A complex. The number shown in the brackets refer to the residue number in the indicated subunit. Inset: Structure of the NALCN-FAM155A interaction interface. K1377 of NALCN is shown in stick. The C-terminal residue in the resolved FAM155A structure is E382, which is about 27 Å from K1377 of NALCN. K395 of FAM155A, 13 residues after E382, is likely to be in the vicinity of cross-linking range with K1377 of NALCN. The cross-linking MS result is consistent with our structure observation.

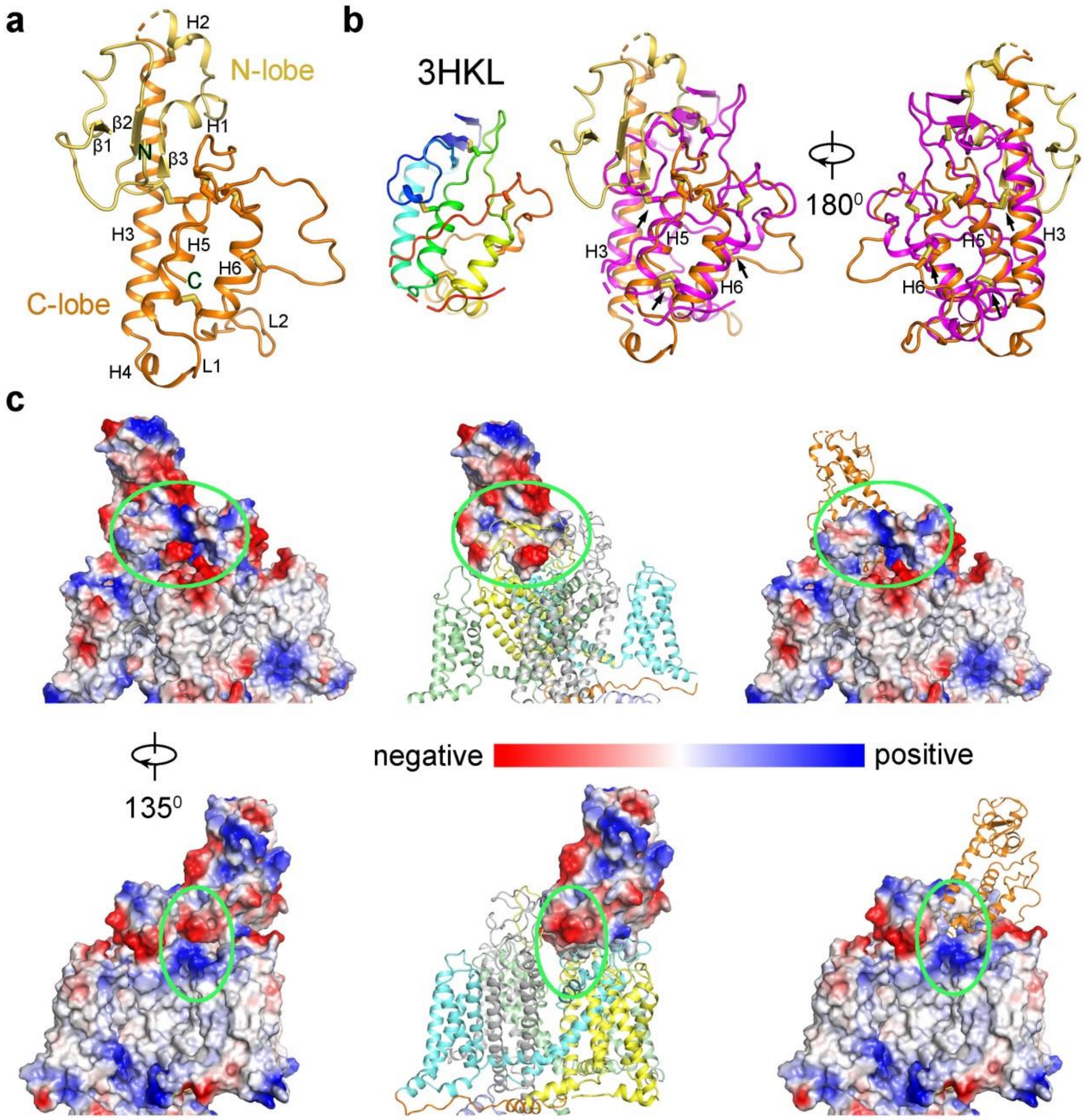

Supplementary Fig. 7. Interaction interfaces between NALCN and FAM155A. (a) Structure of the FAM155A. The N-lobe and C-lobe were colored gold and orange, respectively. The secondary elements are labeled based on the definition in Fig. 3. Disulfide bonds are shown in stick. **(b)** Crystal structure of the CRD domain of MuSK (PDB: 3HKL) and its superimposition with FAM155A. The conserved disulfide bonds between the two structures are highlighted by black arrows. **(c)** Electrostatic surface of the interaction interfaces between NALCN and FAM155A. The structures are shown with both subunits in electrostatic surface (*left*), with NALCN in cartoon and FAM155A in electrostatic surface (*middle*), or with FAM155A in cartoon and NALCN in electrostatic surface (*right*). Two side views are

presented. The green circles highlight the interaction interfaces that have complementary electrical potential between NALCN and FAM155A.

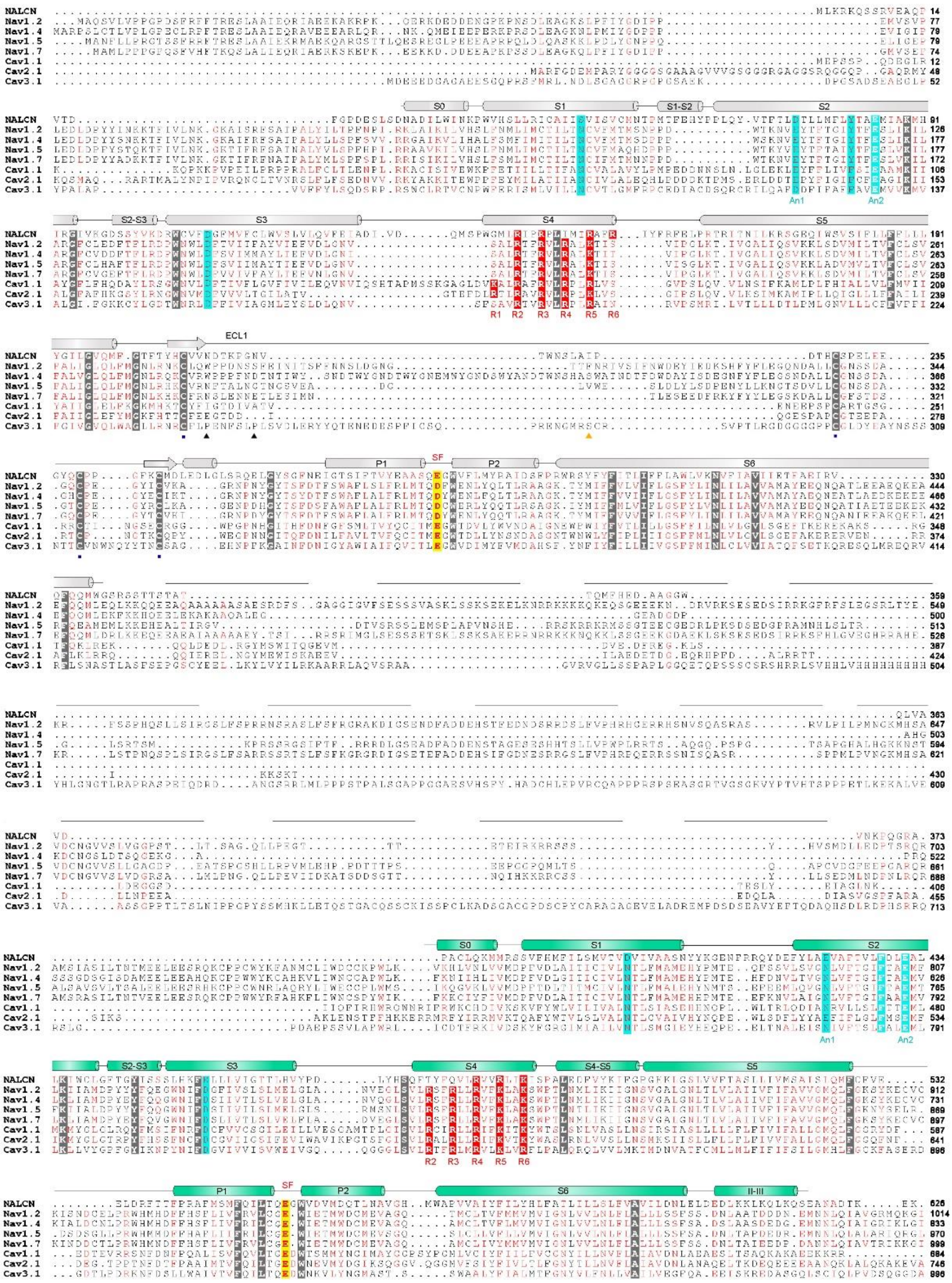
NALCN
Nav1.2
Nav1.4
Nav1.5
Nav1.7
Cav1.1
Cav2.1
Cav3.1
S0
S1
S1-S2
S2
An1
An2
S2-S3
S3
S4
S5
R1 R2 R3 R4 R5 R6
ECL1
P1
SF
P2
S6
S4-S5
R2 R3 R4 R5 R6
II-III

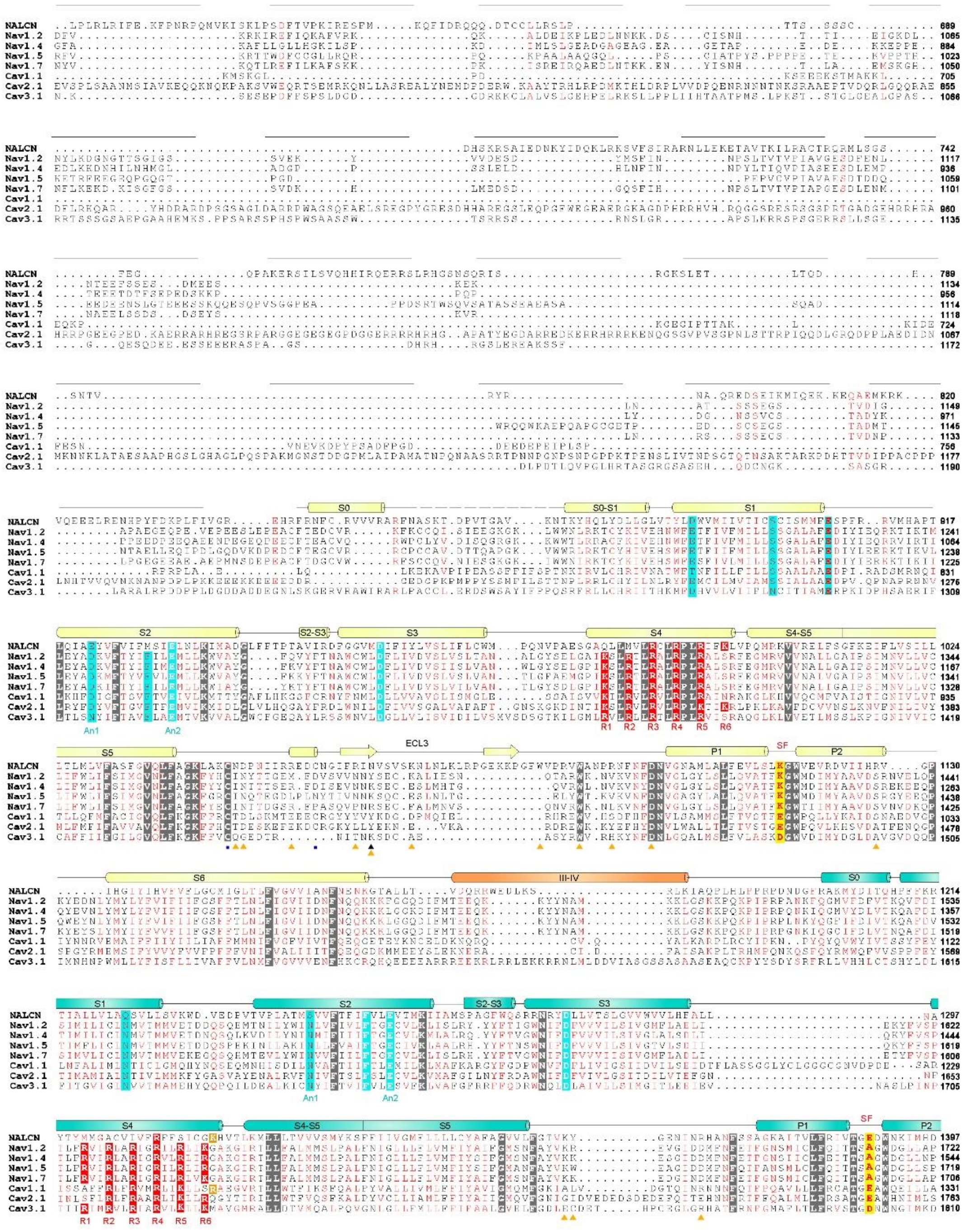

NALCN
Nav1.2
Nav1.4
Nav1.5
Nav1.7
Cav1.1
Cav2.1
Cav3.1
S0
S0-S1
S1
S2
S2-S3
S3
S4
S4-S5
S5
ECL3
P1
SF
P2
S6
III-IV
An1
An2
R1
R2
R3
R4
R5
R6

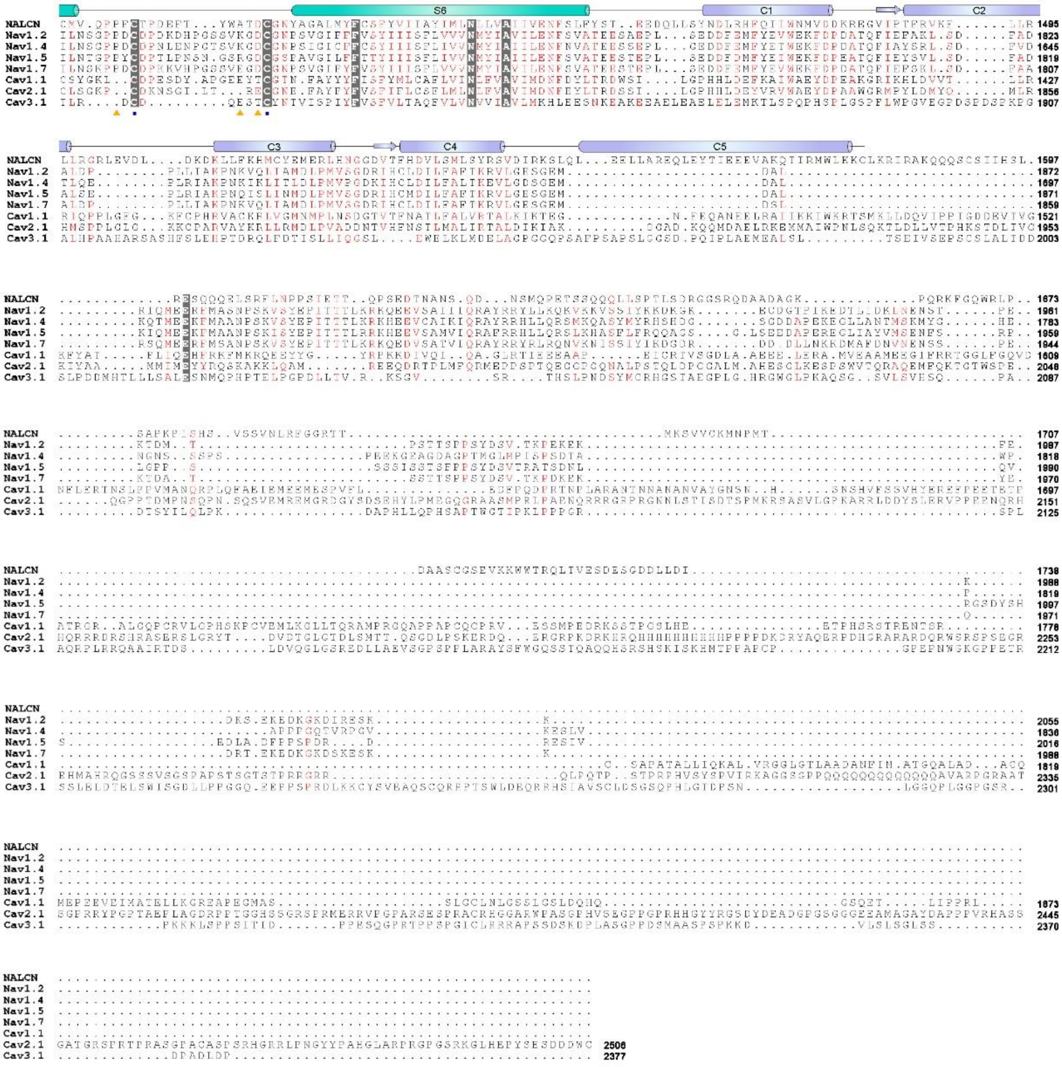

Supplementary Fig. 8 Sequence alignment among NALCN and selected Na_v/Ca_v channels. The primary sequences of four human Na_v subtypes and three human Ca_v subtypes are compared with that of human NALCN using Clustal W (*8*). The invariant residues are shaded gray and the conserved residues are colored red. Secondary structural elements of human NALCN are presented above the sequence alignment and color-coded for each repeat. The critical EEKE residues in the selectivity filter are colored red and shaded yellow. The gating charge residues (labeled R1-R6) in each S4 segments are colored white and shaded red. The residues on S1-S3 that may facilitate the gating charge transfer, including the labeled An1 and An2 sites and the occluding residues on S2, are shaded cyan. The residues that form disulfide bonds are labeled with squares below the sequence alignment. The

glycosylation sites are labeled with black triangles and the residues that mediate interaction with FAM155A are labeled with orange triangles. The Uniprot IDs for the aligned sequences are: hNALCN: Q8IZF0; hNa$_v$1.2:Q99250; hNa$_v$1.4:P35499; hNa$_v$1.5:Q14524; hNa$_v$1.7:Q15858; hCa$_v$1.1: Q13698; hCa$_v$2.1: O00555; hCa$_v$3.1: O43497.

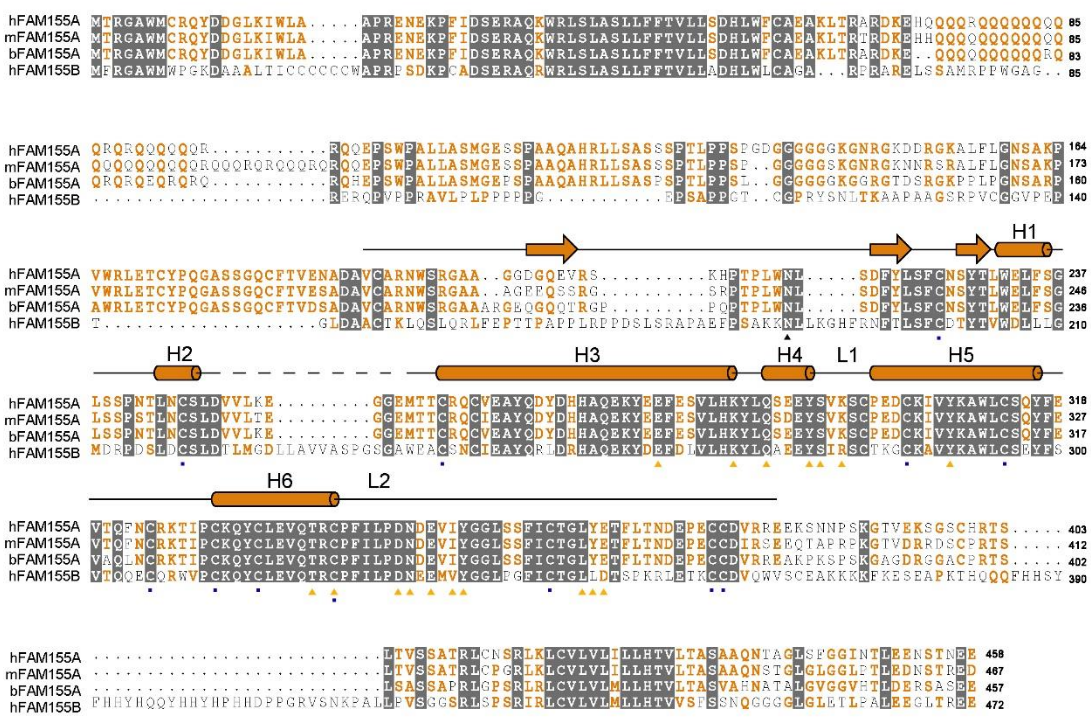

Supplementary Fig. 9. Sequence alignment of FAM155A. The primary sequences of FAM155A from human, mouse, and bovine and FAM155B from human are aligned using Clustal W. The invariant residues are colored white and shaded gray. The conserved residues are colored orange. The secondary structure elements of human FAM155A are indicated above the sequence alignment. The residues that form disulfide bonds are labeled with squares below the sequence alignment. The glycosylation site is labeled with black triangle and the residues that mediate interaction with NALCN are labeled with orange triangles. The Uniprot IDs for the aligned sequences are: hFAM155A: B1AL88, mFAM155A: Q8CCS2; bFAM155A: A4IFM1, hFMA155B:O75949.

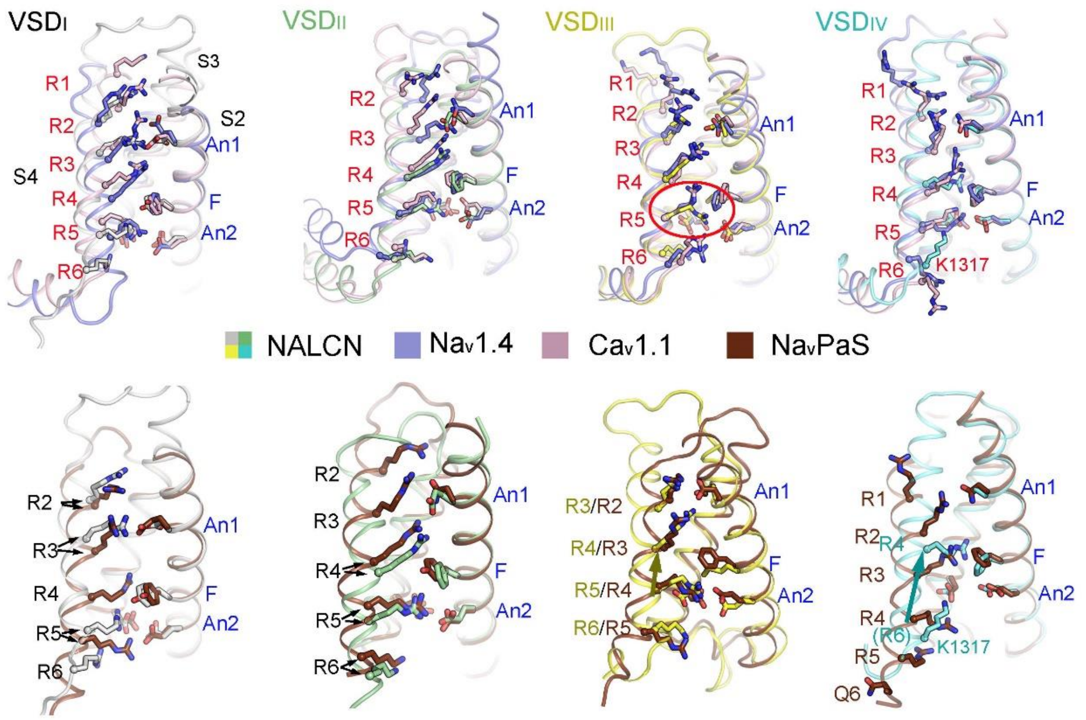

Supplementary Fig. 10. All four VSDs adopt up conformations. Each VSD of NALCN is superimposed with their counterpart in Na_v1.4 (PDB: 6AGF) and Ca_v1.1 (6JP5), or Na_vPaS (6A95). The VSDs are aligned relative to CTC residues and An1 on S2 (labeled blue). S1 segments were omitted for visual clarity. The gating charges (GCs), charge transfer center (CTC) and An1 on S2 are shown as stick and Cα atoms of the GCs are shown as spheres. The GCs on S4 are labeled as R1-R6. Except for R5 on $S4_{III}$ (red circle), the positions of all other GCs in NALCN are similar as that of Na_v1.4 and Ca_v1.1, which represent up or depolarized conformations. By contrast, the positions of GCs in VSD_{III} and VSD_{IV} show an upward shift (brown or dark green arrow) compared to that of Na_vPaS, whose $VSD_{III/IV}$ may represent resting state.

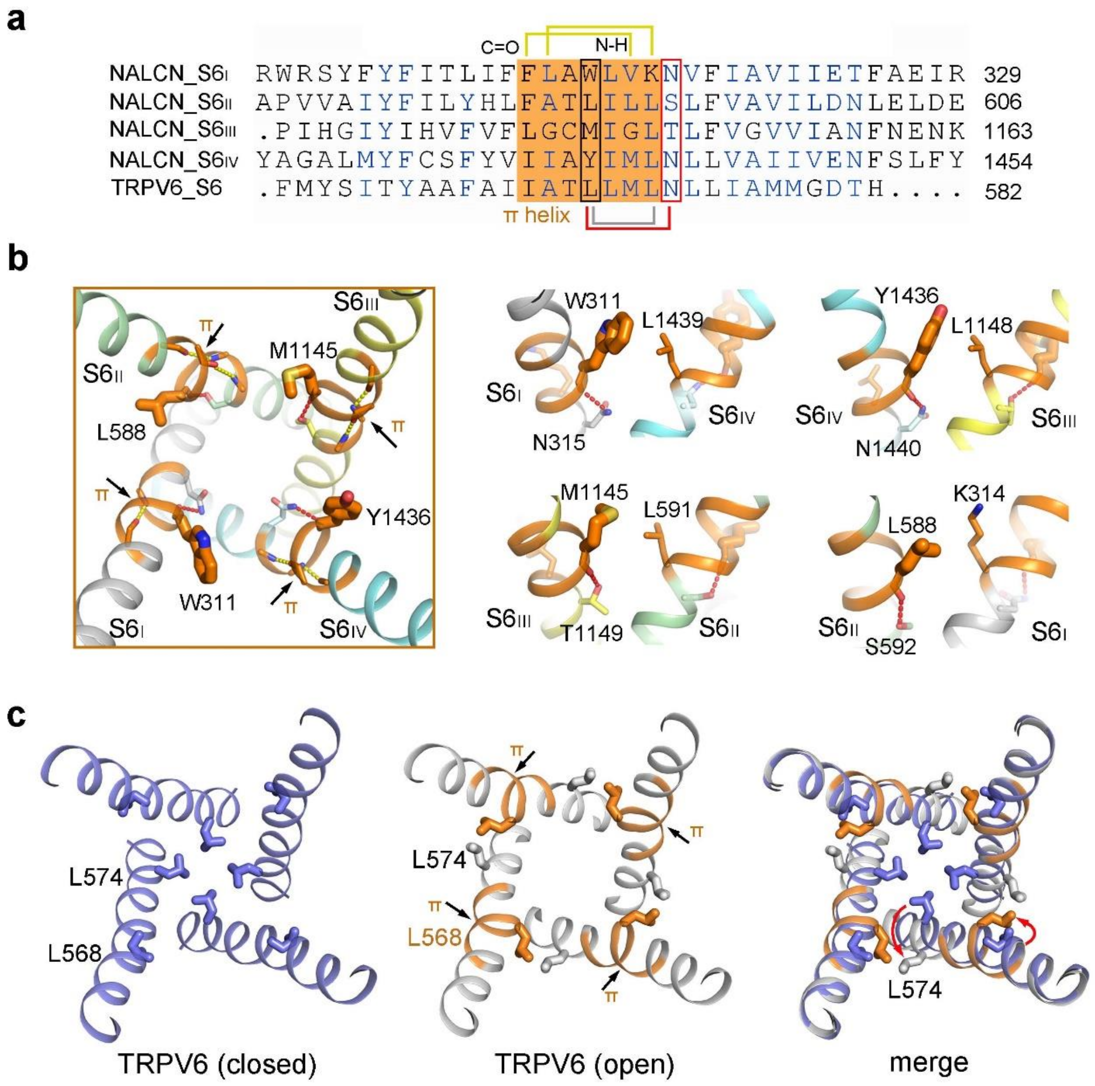

Supplementary Fig. 11. π helices in the S6 segments. (a) Sequence alignment among the S6 segments of NALCN and TRPV6. The conserved residues are colored blue. The seven residues in each S6 that form π helix are shaded orange. Within the π helix, the hydrogen bonds formed by backbone carbonyl group of residues (i) and the amino group of residues (i+5) are indicated by yellow lines. The red line indicates the hydrogen bond formed by the carbonyl group of the residues (i) outlined in a black box with side chains of the residues (i+4) outlined by a red box. The gray line indicates inter repeat interactions. **(b)** Local interactions that stabilize the π helix in S6 segments. Hydrogen bonds are shown in yellow and red dashed lines, as consistent with labels in **(a)**. The residues (i) indicated by a black box in **(a)** are shown in stick. They interact with both the residue (i+4) within the same S6 through hydrogen bonds

and with the residues (i+3) of the adjacent S6 through hydrophobic interactions. The latter interactions fill the pore fenestrations of NALCN. **(c)** Structural comparison of TRPV6 in closed and open states. The S6 segments in the closed state is formed by α helices. The open structure of TRPV6 forms a π helix in S6, in a position that is conserved with that of NALCN. The transition between α helix to π helix induces rotational shifts of the subsequent residues in the S6 segments (red arrows), resulting state changes of the channel.

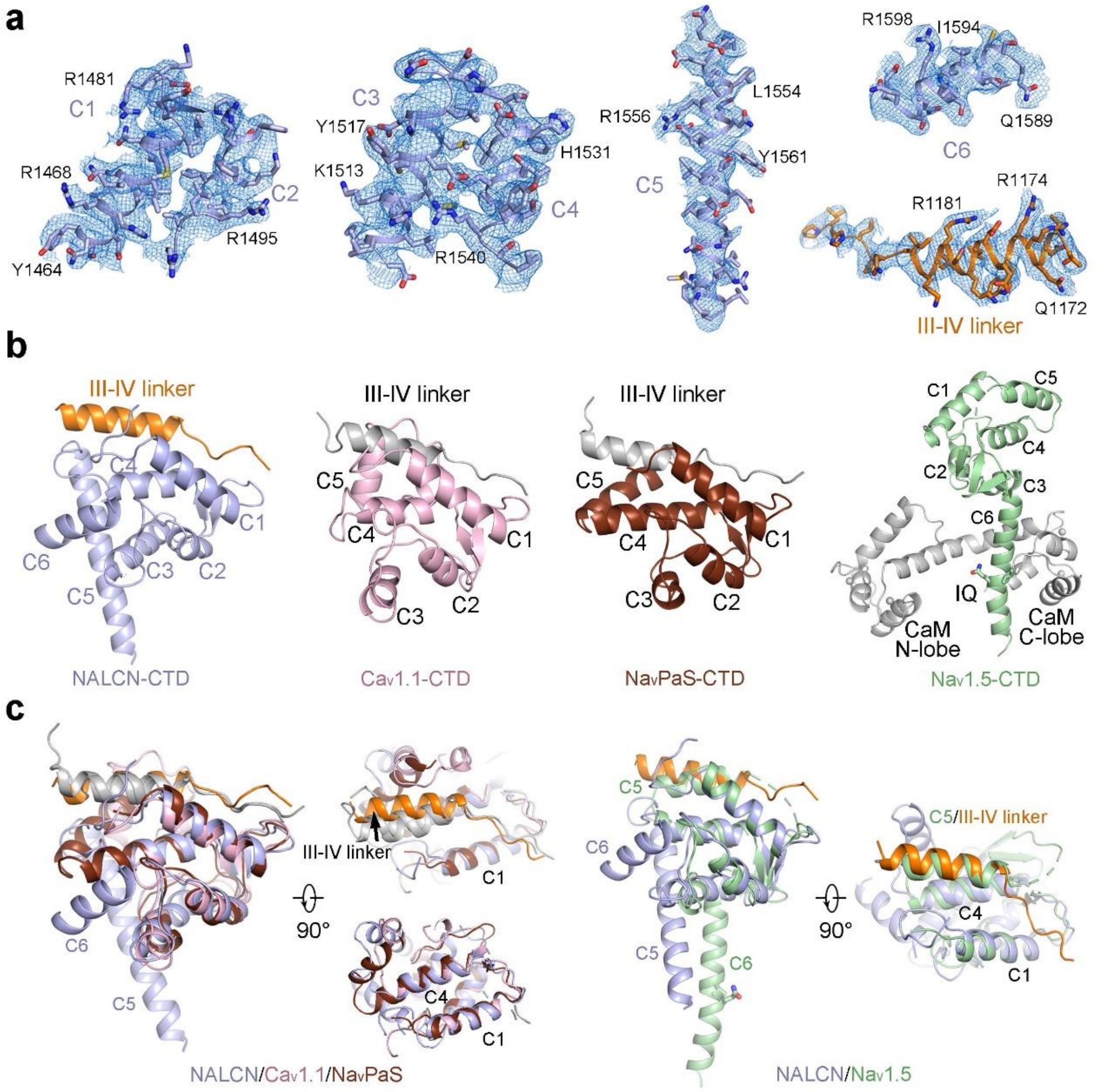

Supplementary Fig. 12. Structural analysis of NALCN CTD. (a) EM densities of each segment of CTD and III-IV linker. All the densities are contoured at 4 σ in PyMol. **(b)** The CTDs of NALCN, Ca_v1.1 (PDB: 6JP5), Na_vPaS (PDB: 6A95), and Na_v1.5 (PDB: 6MUD) all share a similar globular fold. Na_v1.5-CTD is a crystal structure of the single domain, while the others are part of the full length cryo-EM structure. The interacting III-IV linker or calmodulin (CaM) are also shown as cartoon. The IQ motif in the C-terminal of Na_v1.5 is shown in stick. **(c)** Structure comparison of the CTDs. C1-C4 helices of CTD of NALCN fit well with that of other CTDs, but C5-C6 are distinct. When the CTDs are superimposed, the III-IV linker of NALCN displays a shift compared to that of Ca_v1.1 and Na_vPaS, as indicated by a black arrow (*left*); or superimposes with C5 helix with that of Na_v1.5 (*right*).

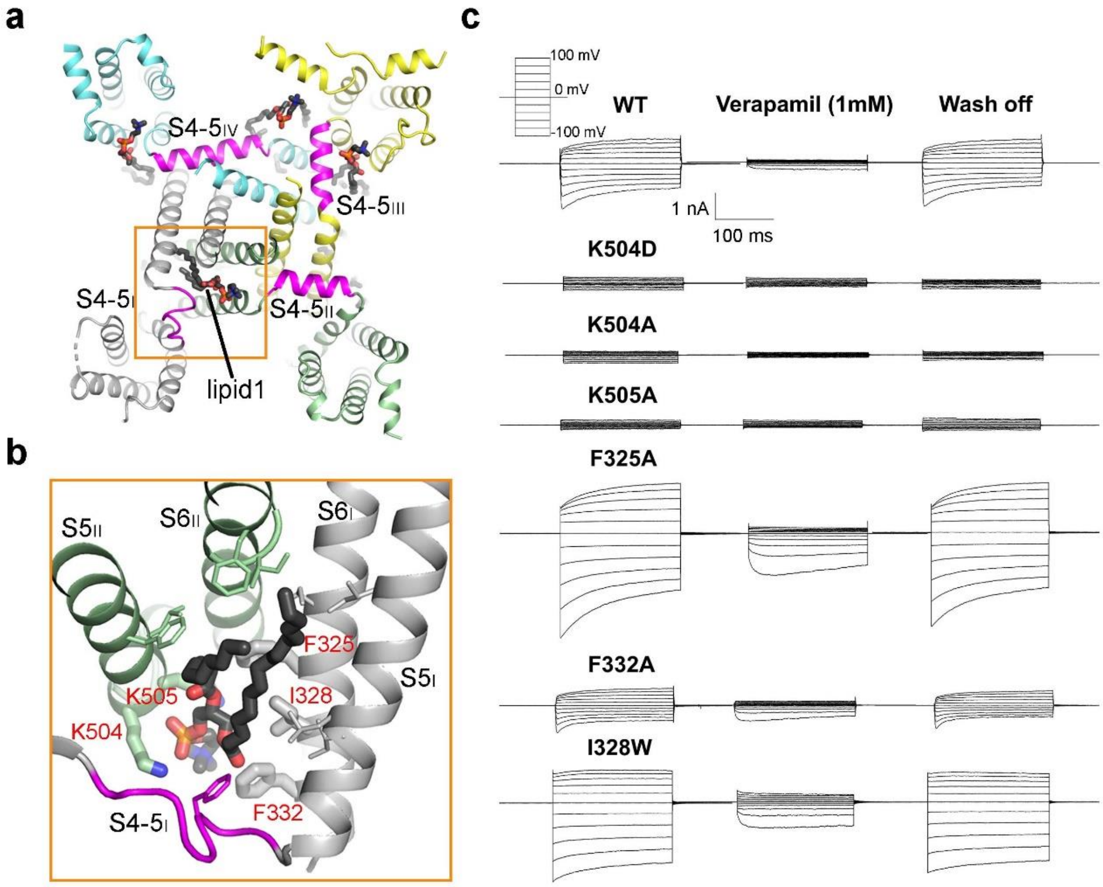

Supplementary Fig. 13. A unique lipid that may play a role in NALCN function. **(a)** The identified lipid molecules in the intracellular side of NALCN are all close to the S4-5 linkers. While other lipids bind in the semi-closed clefts formed by S4-5 linker and adjacent VSD, lipid1 (indicated by a square box) sits in a closed cavity surrounded by S4-5_I loop and S5, S6 segments of repeat I and repeat II. **(b)** Detailed interaction between lipid1 and NALCN. The tail group of lipid1 is surrounded by many hydrophobic residues. Two positively charged residues K504 and K505 could form hydrogen bond with the head group of lipid1. The residues that are mutated for functional studies are shown in stick. **(c)** Mutants in the interaction interface between lipid1 and NALCN can affect the function of NALCN. Shown here are the representative whole-cell recordings of NALCN and mutants in the presence of FAM155A, UNC79, and UNC80 in HEK293 cells. NALCN inhibitor verapamil was added and then washed off during the recording.

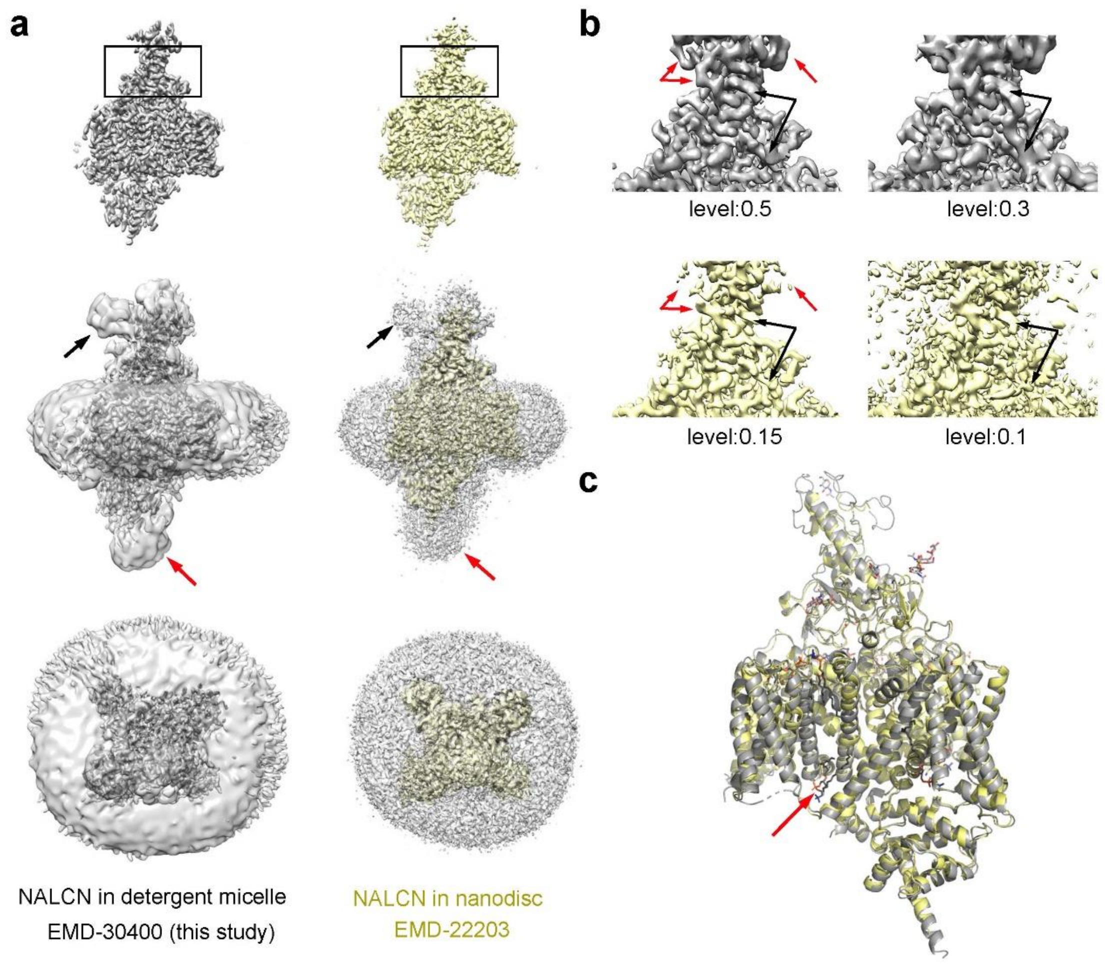

Supplementary Fig. 14. Comparison of NALCN structures in detergent micelle or in nanodisc. (a) The overall cryo-EM maps of NALCN are similar, although the sample were prepared in different conditions. When the maps were contoured at low levels, some extra densities that may belong to FAM155A and CTD were observed in both cases, indicated by black and red arrows, respectively. The size of GDN detergent micelle is slightly larger than that of nanodisc. Details in the rectangle boxes are presented in **(b)**. **(b)** The map in this study shows better quality in some local regions, indicated by red arrows. Each map is shown in two contour levels in Chimera for better comparison. A density that belongs to the FAM155A (365-375) is continuous in the map of this study, indicated by black arrows. **(c)** The overall structures solved in two different conditions are similar, with an r.m.s.d. of 0.7 Å when superimposed. The unique lipid identified in our study is indicated by a red arrow.

References

1. R. Fukai *et al.*, De novo missense mutations in NALCN cause developmental and intellectual impairment with hypotonia. *J Hum Genet* **61**, 451-455 (2016).
2. N. C. Bramswig *et al.*, Genetic variants in components of the NALCN-UNC80-UNC79 ion channel complex cause a broad clinical phenotype (NALCN channelopathies). *Hum Genet* **137**, 753-768 (2018).
3. J. X. Chong *et al.*, De novo mutations in NALCN cause a syndrome characterized by congenital contractures of the limbs and face, hypotonia, and developmental delay. *Am J Hum Genet* **96**, 462-473 (2015).
4. M. Karakaya *et al.*, Novel Mutations in the Nonselective Sodium Leak Channel (NALCN) Lead to Distal Arthrogryposis with Increased Muscle Tone. *Neuropediatrics* **47**, 273-277 (2016).
5. M. D. Al-Sayed *et al.*, Mutations in NALCN cause an autosomal-recessive syndrome with severe hypotonia, speech impairment, and cognitive delay. *Am J Hum Genet* **93**, 721-726 (2013).
6. C. Koroglu, M. Seven, A. Tolun, Recessive truncating NALCN mutation in infantile neuroaxonal dystrophy with facial dysmorphism. *J Med Genet* **50**, 515-520 (2013).
7. D. K. Bourque *et al.*, Periodic breathing in patients with NALCN mutations. *J Hum Genet* **63**, 1093-1096 (2018).
8. M. A. Larkin *et al.*, Clustal W and Clustal X version 2.0. *Bioinformatics* **23**, 2947-2948 (2007).
