# Supplementary material for: Structure of the human sodium leak channel NALCN in complex with FAM155A
Source: Nat Commun. 2020 Nov 17;11:5831. doi: 10.1038/s41467-020-19667-z (PMC7672056; doi:10.1038/s41467-020-19667-z)
Supplement: Supplementary file 3 — Description of Additional Supplementary Files [file 41467_2020_19667_MOESM3_ESM.pdf]

Description of Additional Supplementary Files

File name: Supplementary Movie 1
Description: MD simulation of NALCN pore domain for identification of sodium binding sites. Left panel. Top view of selectivity filter. Ion binding residues are highlighted in stick representation. Probability density of sodium is illustrated in mesh grid. Sodium ions are colored in magenta. Right panel. Side views of selectivity filter.
